# Response of turkey muscle satellite cells to thermal challenge. I. transcriptome effects in proliferating cells

**DOI:** 10.1186/s12864-017-3740-4

**Published:** 2017-05-06

**Authors:** Kent M. Reed, Kristelle M. Mendoza, Juan E. Abrahante, Natalie E. Barnes, Sandra G. Velleman, Gale M. Strasburg

**Affiliations:** 1 Department of Veterinary and Biomedical Sciences, University of Minnesota, St. Paul, MN USA; 2 University of Minnesota Informatics Institute, University of Minnesota, Minneapolis, MN USA; 3 Department of Animal Sciences, The Ohio State University, Columbus, OH USA; 4 Ohio Agricultural Research and Development Center, Wooster, OH USA; 5 Department of Food Science and Human Nutrition, Michigan State University, East Lansing, MI USA

**Keywords:** Satellite cell, Skeletal muscle, Growth selected, Turkey, Proliferation

## Abstract

**Background:**

Climate change poses a multi-dimensional threat to food and agricultural systems as a result of increased risk to animal growth, development, health, and food product quality. This study was designed to characterize transcriptional changes induced in turkey muscle satellite cells cultured under cold or hot thermal challenge to better define molecular mechanisms by which thermal stress alters breast muscle ultrastructure.

**Results:**

Satellite cells isolated from the pectoralis major muscle of 7-weeks-old male turkeys from two breeding lines (16 weeks body weight-selected and it’s randombred control) were proliferated in culture at 33 °C, 38 °C or 43 °C for 72 h. Total RNA was isolated and 12 libraries subjected to RNAseq analysis. Statistically significant differences in gene expression were observed among treatments and between turkey lines with a greater number of genes altered by cold treatment than by hot and fewer differences observed between lines than between temperatures. Pathway analysis found that cold treatment resulted in an overrepresentation of genes involved in cell signaling/signal transduction and cell communication/cell signaling as compared to control (38 °C). Heat-treated muscle satellite cells showed greater tendency towards expression of genes related to muscle system development and differentiation.

**Conclusions:**

This study demonstrates significant transcriptome effects on turkey skeletal muscle satellite cells exposed to thermal challenge. Additional effects on gene expression could be attributed to genetic selection for 16 weeks body weight (muscle mass). New targets are identified for further research on the differential control of satellite cell proliferation in poultry.

**Electronic supplementary material:**

The online version of this article (doi:10.1186/s12864-017-3740-4) contains supplementary material, which is available to authorized users.

## Background

Climate change poses a multi-dimensional threat to food and agricultural systems by affecting plant and animal production systems, stability of food supplies, food quality and access to food [1]. In addition to the increase in mean temperature, there is a predicted increase in the frequency of extreme temperature days [2, 3]. Such volatile temperatures put animals at increased risk of thermal stress, thereby potentially affecting animal growth, development, health, and food product quality.

Thermal stress can manifest as extremes of both hot and cold temperatures. In addition to the obvious welfare issues, the effects of hot temperatures have been of particular interest to poultry producers due to effects on muscle that reduce meat quality. Specifically, in chickens, chronic heat exposure results in lower weight gain, lower ratio of breast muscle to body weight, and increased intramuscular fat deposition [4]. Turkey meat in particular is valued for its low fat content, thus increased fat deposition can decrease meat quality. In contrast, relatively severe cold stress slightly before slaughter in chickens resulted in decreased meat quality via changes in pH, color, and drip loss [5]. Moderate cold stress in young chickens has also been shown to cause damage to the heart and duodenum – with the severity of injury increasing with the length of exposure [6, 7]. The nature and extent of the physiological response to thermal stress is a function of genetic background as well as the timing of the stress [8, 9]. Fast growing lines of poultry tend to be more sensitive to thermal stress than slower growing lines [9].

In this study, we utilized cultured turkey satellite cells as a model system to study the effects of thermal stress on muscle growth and development. Satellite cells are muscle stem cells located between the basement membrane and sarcolemma of skeletal muscle fibers [10]. In the early post-hatch period, avian satellite cells respond to mild heat stress with accelerated proliferation and differentiation [11]. Evidence from cell culture studies suggest that satellite cells are multi-potential stem cells which can be induced to follow myogenic, osteogenic, or adipogenic cellular pathways [12, 13]. Satellite cells are highly active during the period of rapid growth after hatch [11] and then become quiescent until they are needed for hypertrophy or repair [14]. The increased fat deposition noted above may be the result of transdifferentiation of muscle satellite cells into adipocytes.

This study seeks to characterize the transcriptional changes induced in satellite cells cultured under hot or cold thermal challenge to better define molecular mechanisms by which thermal stress alters turkey breast muscle ultrastructure and consequently food quality. Here we compare gene expression between cultured satellite cells derived from both fast and slow-growing lines in the context of thermal challenge. We hypothesize that satellite cell activity, its ultimate fate as a muscle cell or adipocyte, and thus overall muscle development will be altered by temperature. Determining the satellite cell-mediated mechanisms associated with the development of superior meat quality will allow development of effective breeding, nutritional, and management strategies to promote the production of consistent, high quality muscle food products.

## Methods

### Turkey myogenic satellite cells

Satellite cells were isolated from the pectoralis major muscle of 7-weeks-old males from the Randombred Control 2 (RBC2) and body weight-selected (F) turkey lines as previously described [15]. The RBC2 line is maintained at the Poultry Research Center of the Ohio Agricultural Research Development Center/The Ohio State University Wooster, OH without conscious selection for any trait [16]. The F line was derived from the RBC2 line and selected only for 16weeks body weight [16, 17]. These larger and faster- growing turkeys have greater p. major muscle and body weights than the RBC2 line [18, 19].

Turkey p. major satellite cells were plated at a density of 15,000 cells per well in 0.1% gelatin coated 24-well plates (Greiner BioOne, Monroe, NC). After plating, cells were incubated in a 38 °C 95% air/5% CO_2_ incubator (Thermo Fisher Scientific, Pittsburgh, PA) for 24 h in plating medium consisting of Dulbecco’s Modified Eagle’s Medium (DMEM, Sigma Aldrich, St. Louis, MO), 10% chicken serum (Gemini BioProducts, West Sacramento, CA), 5% horse serum (Gemini BioProducts), 1% antibiotics-antimycotics, and 0.1% gentamicin. After 24 h the plating medium was removed and the cells were fed growth medium consisting of McCoy’s 5A (Sigma Aldrich), 10% chicken serum, 5% horse serum, 1% antibiotics-antimycotics, and 0.1% gentamicin. Cells were cultured in a 38 °C 95% air/5% CO_2_ incubator (control) or in a 95% air/5% CO_2_ incubator held at an experimental temperature (33° or 43 °C) for 72 h. Growth medium was changed every 24 h for the 72 h treatment. Cell medium was removed and the plates were held at −80 °C until RNA isolation. The control temperature of 38 °C is lower than the body temperature of mature turkeys (41.5 °C) but is approximately that measured in newly hatched poults (38.0–38.5 °C, Strasburg, unpubl). Growth characteristics of these cells are further detailed in Clark et al. [20].

### RNA isolation and sequencing

Total RNA was isolated from each sample by TRIzol extraction (Ambion, Inc.), DNase-treated (Turbo DNA-*free* ^TM^ Kit, Ambion, Inc.), and stored at −80 °C. Initial RNA concentration and quality was assessed by spectrophotometry (Nanodrop 1000). RNA samples were submitted for library preparation and sequencing at the University of Minnesota Genomics Center. Each sample was quantified by RiboGreen Assay (Invitrogen Corp.) and RNA integrity was confirmed on the 2100 Bioanalyzer (Aligent Technologies). Replicate samples were sequenced from each treatment group (*n* = 12). Each sample had clear 18S and 28S peak separation on the electropherograms and RNA Integrity Number (RIN) between 6.4 and 8.5. Indexed libraries were constructed with 1 μg of total RNA/sample with the TruSeq RNA Sample Preparation Kit version 2 (Illumina, Inc.) and size selected for approximately 200 bp inserts. Libraries were multiplexed, pooled and sequenced over 2 lanes on the HiSeq 2000 using v3 chemistry (Illumina, Inc.) to produce 101-bp paired-end reads.

### RNAseq data analyses

Sequence adapters were removed and low quality bases were trimmed using Trimmomatic [20] enabled with the optional “-q” option; 3 bp sliding-window trimming from 3’ end requiring minimum Q30. Quality control checks on raw sequence data for each sample were performed with FastQC [21]. Read mapping was performed via Bowtie (v2.2.4.0) using the turkey genome (UMD 5.0, NCBI Annotation 101). Read counts were normalized in CLC Genomics Workbench (CLCGWB v. 8.0.2, CLC Bio) by dividing the total read counts by the group sample sum and the results expressed as reads per 16M. Hierarchical clustering of samples (based on Euclidean sample distances with single linkage) was performed in CLCGWB using normalized reads counts. Empirical analysis of differential gene expression and ANOVA was performed in CLCGWB on original expression values (Bonferroni and FDR corrected). Principal component analysis (PCA) was performed in CLCGWB to identify and quantify variability in the data. Volcano plots and Venn diagrams were used to visualize the expression data and the results of significance testing. Pair-wise comparisons between treatment groups were made in the Bioconductor (3.2) R package DESeq2 [22] following the standard workflow. In each pair-wise comparison, significant DE genes were used to investigate affected gene pathways using Ingenuity Pathway Analysis (IPA) (Ingenuity Systems, Redwood City, CA). Gene enrichment tests were performed using the PANTHER Overrepresentation Test (GO Consortium release 20150430, [23]; http://geneontology.org/). GO analysis utilized the reference gene set of the chicken (*Gallus gallus*) of which ~63% of the turkey loci (Annotation 101) had ID homologs.

## Results

Total RNA isolated from satellite cell cultures (*n* = 12) was used for construction of individual barcoded libraries. Sequencing of all libraries produced over 195M reads (accessioned as part of SRA BioProject 341399). The number of reads per library ranged from 12.4M to 18.5M (average 16.3M) (Table 1). After read trimming and filtering, median Q score was consistently high and ranged from 36.8 to 37.3. Box-plots demonstrate that quality scores across base position in each corrected dataset were sufficiently high for reliable base calling (Additional file 1: Figure S1). The number of reads per treatment group ranged from 14.5 to 17.2M with an average of 16.28M ± 1.06 M reads. Replicate libraries produced comparable results with an average difference between replicates of 1.6M reads.

**Table 1:** Summary of RNAseq data for proliferation experiment

Line	Temp °C	Replicate	PE reads	Median read quality R1	Median read quality R2	Trimmed PE reads	% mapped	% concordant	Estimated insert mean (bp)	Total observed genes	Mean expressed genes	% expressed genes
RBC2	33	A	18492232	37.3	37.2	17160507	90.0	84.3	199	16527	15065	71.7
		B	15688650	37.3	37.2	14526144	89.8	84.0	199	16305		
	38	A	17462871	37.1	36.9	15938819	88.3	81.6	227	16408	14524	69.1
		B	16861464	37.1	36.9	15388173	88.4	81.8	226	16186		
	43	A	12482369	37.1	36.8	11301872	88.4	82.2	194	15864	14241	67.8
		B	16552504	36.9	36.8	15135313	87.3	80.5	199	15864		
F-Line	33	A	15640211	37.2	37.1	14477399	89.7	84.0	199	15734	15091	71.8
		B	15574971	37.2	37.1	14421868	89.6	83.5	199	15814		
	38	A	15615066	37.2	37	14320803	88.5	81.9	218	15343	14586	69.4
		B	16932717	37.1	36.9	15464075	89.1	82.5	226	15365		
	43	A	16624565	37.1	36.8	15156088	89.1	81.9	225	15886	14771	70.3
		B	17476215	37.1	37	16091169	89.2	82.8	198	15874		
Mean			16283652.9	37.14	36.98	14948529.2	88.95	82.58	209.1	15930.8	14713	70.03

For each library the total number of concatenated reads, median read qualities (R1 and R2), estimated mean insert length (bp), number of and percentage of aligned reads, percentage of concordant reads, and the number and percentage of observed genes (mapped reads >1) and expressed genes (mean group normalized read count >3.0) are given

### Gene expression

Approximately 89% of the quality trimmed reads mapped uniquely to the annotated turkey gene set (Table 1) with an estimated mean library insert of 209.1 bp. This percentage was consistent across treatment groups. For all libraries, the percentage of aligned read pairs exceeded 87% (avg 88.95%) and the majority of reads (avg 82.6%) mapped concordantly to the gene set (Table 1). A total of 16,515 genes was detected (minimum one mapped read in at least one treatment group) with an average of 15,930 per treatment group (74.3% of the turkey gene set). Mean read depth was 491.3 reads/gene (Additional file 2: Table S1). When limited by the average number of mapped reads (≥3.0), the mean number of expressed genes (tRNAs excluded) was reduced (average 14,712) and ranged from 12,192 to 13,094 within treatment groups (Table 1).

**Fig. 1:**
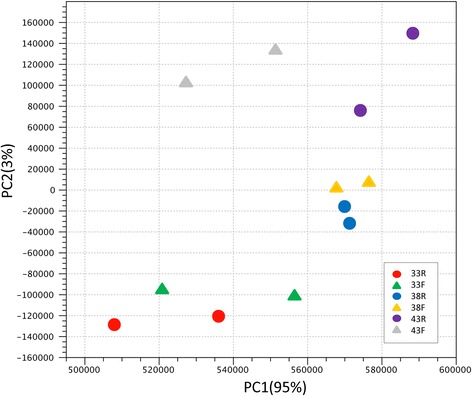
Principal component analysis (PCA) of RNAseq data based on normalized read counts. Sample to sample distances (within- and between-treatments) are illustrated for each dataset on the first two principal components comprising approximately 98% of the variation. Samples are plotted according to treatment

Principal component analysis (PCA) was used to visualize the variation among treatment groups based on normalized read counts (Fig. 1). Treatment groups generally clustered distinctly (Temp/Time) within the first two principal components (explaining 98% of the observed variation) irrespective of line. Hierarchical clustering of groups by Euclidean distance reiterated the relationships shown by PCA (Additional file 1: Figure S2). Groups clustered by line and incubation temperature. All replicate treatment pairs occurred as nearest neighbors, supporting the pooling of replicates for expression analyses. Differences in gene expression among groups are illustrated in the heat map which includes the co-expressed genes with the greatest experiment-wise differences in average expression.

Distribution of unique and shared expressed genes among treatment groups are illustrated in Table 2. On average 14,245 genes were co-expressed between treatment groups, within temperature and 13,794 genes were co-expressed between lines. This reflects an overall similarity in response of cultured satellite cells between the turkey lines. Numbers of uniquely expressed genes were higher for the F-line at all incubation temperatures (453, 449, and 809 at 33 °C, 38 °C and 43 °C, respectively). In comparing treatment groups, the highest numbers of shared genes occurred between the 33 °C and 38 °C treatments for both lines (Table 2).

**Table 2:** Summary of gene expression and significant differential expression (DE) in pair-wise comparisons of proliferating cells

Comparison	Groups	Total expressed genes	Shared genes	Unique Genes in each group	FDR < 0.05	|Log_2_FC| > 1.0	|Log_2_FC| > 2.0
Cold	33R v 38R	15341	14248	817/276	8826	2746 (0.650)	676 (0.782)
	33F v 38F	15457	14220	871/366	8727	3151 (0.693)	1048 (0.768)
Hot	43R v 38R	14896	13869	372/655	1916	1028 (0.459)	264 (0.417)
	43F v 38F	15238	14119	652/467	4596	1386 (0.674)	495 (0.770)
Line							
	33F v 33R	15518	14638	453/427	2092	412 (0.464)	114 (0.403)
	38F v 38R	14973	14137	449/387	2122	263 (0.319)	72 (0.250)
	43F v 43R	15050	13962	809/279	1172	784 (0.764)	320 (0.859)

For each comparison of the treatment groups (Temperature, 33°, 38° or 43 °C/Line, RBC2 or F), the total number of expressed and uniquely expressed genes, number of genes with significant FDR p-value, and the numbers of significant genes also with |Log_2_ fold change| >1.0 and >2.0 are given. Only those genes with treatment group mean normalized read counts >3.0 are included as expressed. Numbers in parentheses equal the proportion of up-regulated genes

**Fig. 2:**
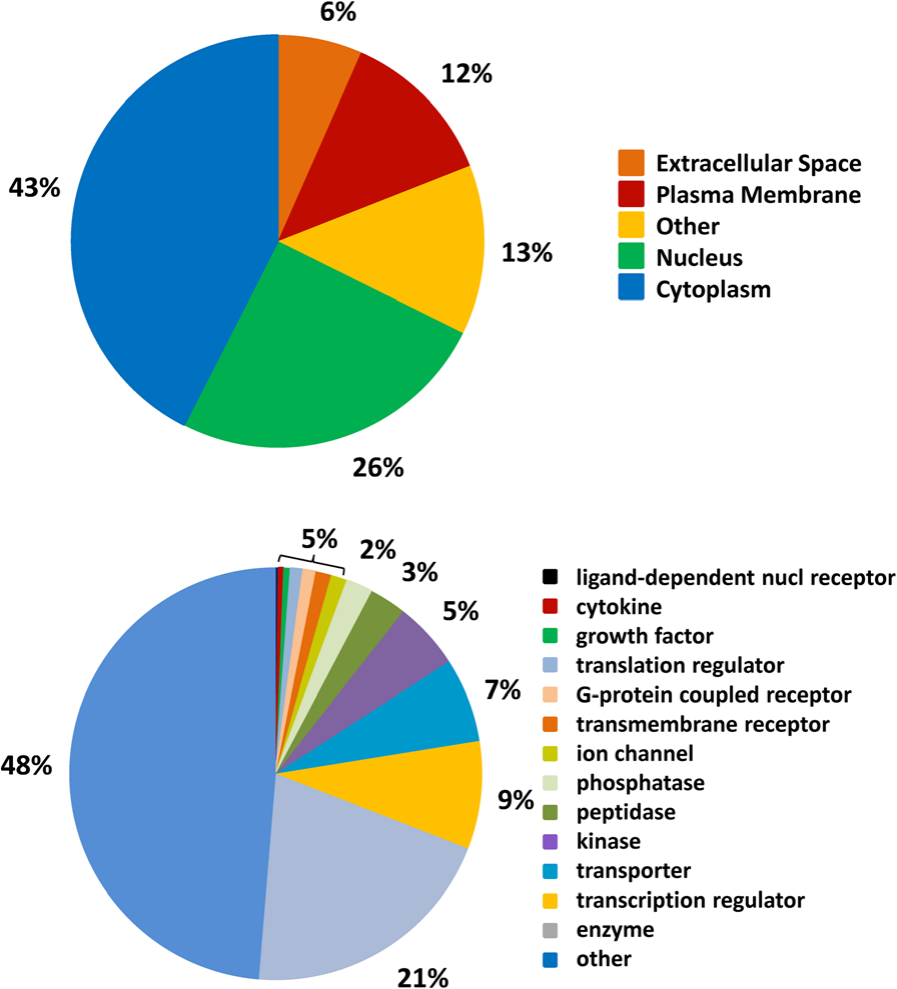
GO classification of genes expressed in cultured turkey p. major satellite cells after 72 h proliferation at 38 °C

Characterization of the expressed genes in satellite cells under the control incubation temperature (38 °C) provided a description of the common cellular processes of these cultured cells (Fig. 2, Additional file 1: Figure S2, Additional file 3: Table S2). At 72 h of proliferation, the majority of gene products (69%) are characterized as nuclear or cytoplasmic proteins. Plasma membrane and extracellular proteins comprise the other major groups (12% and 6%, respectively). Enzyme, transcription regulator, and transporter are the largest represented functional classes of proteins. However, the largest class of gene products (category “other”, 48%) includes primarily structural proteins (Fig. 2).

Of the turkey annotated gene set, 11,615 IDs mapped in the IPA database. Normalized read counts for the 8,000 genes with the highest average read count were used to conduct a Comparison Analysis between the RBC2 and F-line groups at 38 °C. The 20 most significant Metabolic and Signaling pathways identified in the expressed genes are summarized in Additional file 4: Table S3. Most significant in the Metabolic category are tRNA Charging (33 of 39 pathway genes associated) and the Inositol Phosphate Compound superpathway (110 of 193 genes). Significant Signaling pathways include Protein Ubiquitination (179 of 255 genes associated) and EIF2 Signaling (136 of 184 genes).

### Differential expression

**Fig. 3:**
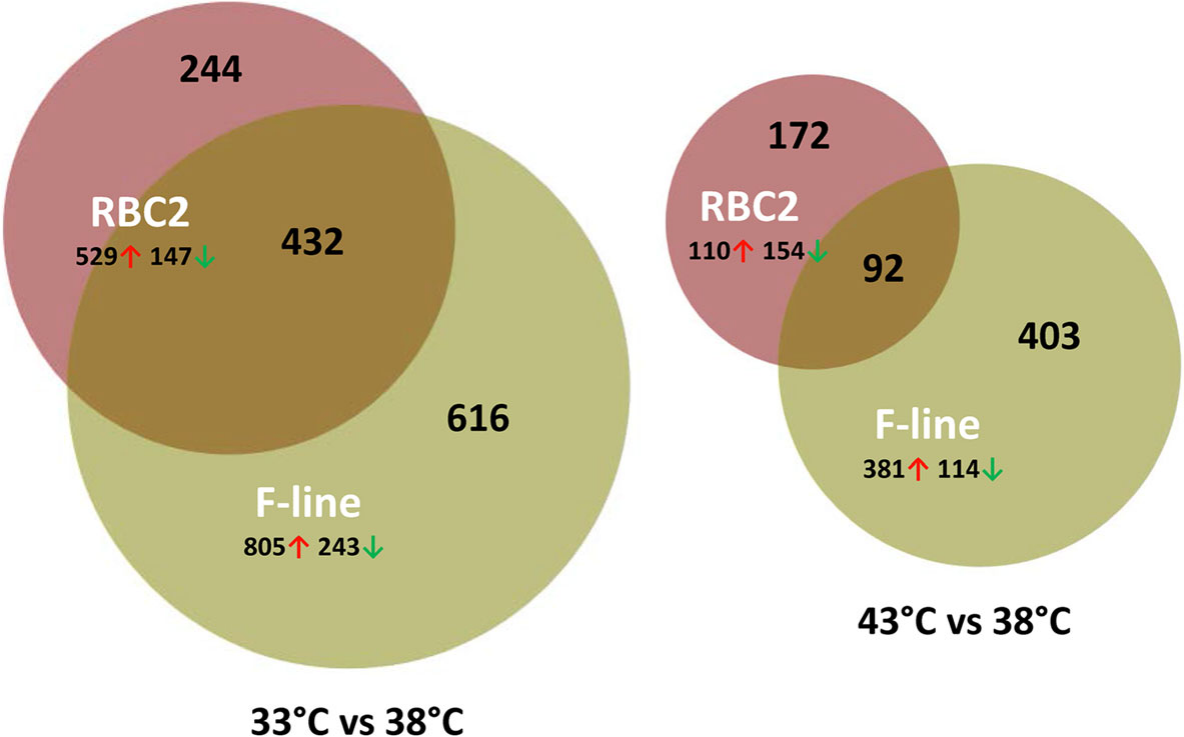
Distribution of differentially expressed genes during proliferation of cultured turkey p. major satellite cells. For each temperature comparison, the number of genes with FDR pval <0.05 and |Log_2_FC| > 2.0 that were shared or unique to each line (RBC2 and F) are indicated in the Venn diagram [71]. Circle size is proportional to the number of genes

Gaussian-based ANOVA found 9,011 genes with significant (FDR p-val <0.05) experiment-wise differential expression (Additional file 1: Figure S3). Seven two-way contrasts were generated based on temperature (cold and hot) and line (RBC2 and F). Large numbers of significant differentially expressed (DE) genes were identified in each contrast (Table 2, Additional file 5: Table S4). A greater number of genes were significantly affected by cold (33 °C) treatment than by hot (43 °C) with more DE genes identified in the F-line at both 33 °C and 43 °C compared to control (Fig. 3). In the temperature comparisons, a greater proportion of DE genes were up-regulated, except in the RBC2 43 °C versus 38 °C comparison where the proportion of down-regulated genes was greater (Table 2, Fig. 3). The 50 significant DE genes with the greatest expression change for each treatment comparison are listed in Additional file 6: Table S5.

Statistical overrepresentation tests (PANTHER, [23]) of genes differentially expressed between the 33 °C and 38 °C found greatest enrichment for the GO Biological Processes of muscle system, synaptic transmission, and cell-signaling (Additional file 7: Table S6). Greatest enrichment of GO Cellular Components included sarcomere, ion channel complex and transmembrane transporter complex whereas calcium ion binding and signal transducer activity represented significantly enriched GO Molecular Functions. In contrast, overrepresentation tests of genes differentially expressed between the 43 °C and 38 °C found significant enrichment for several GO Biological Processes defining muscle development (Additional file 8: Table S7). Seven of these GO categories (synaptic transmission (GO:0007271), regulation of myotube differentiation (GO:0010830), regulation of striated muscle cell differentiation (GO:0051153), muscle contraction (GO:0006936), regulation of muscle contraction (GO:0006937), regulation of muscle system process (GO:0090257), and muscle system process (GO:0003012)) showed greater than 5-fold enrichment. Enrichment of GO Cellular Components reflected the shift towards muscle structure as did Molecular Function.

Although the Ingenuity Knowledge Base of (IPA) is human-centric, it provides useful insight into non-mammalian biological systems. IPA analysis of the turkey DE genes demonstrated several temperature-induced shifts in the satellite cell transcriptomes. Consistent with ongoing cellular development, many of the statistically enriched pathways involved in cell signaling (Additional file 9: Table S8) and the top gene networks involved skeletal and muscle system development. Cell signaling is an import aspect of satellite cells in regulating cell-to-cell interactions during development and homeostasis and in controlling self-renewal. The calcium and calmodulin activated gene *NFATC2* (nuclear factor of activated T cell isoforms C2) was common to several of these pathways. This gene has been shown in mammals to be critical for the fusion of myoblasts with nascent myotubes [24]. Also important were ligands such as the Wnt family of proteins, receptor and modulating molecules such as *FZD* (Fizzled class receptor) and *DKK* (Dickkopf WNT signaling pathway inhibitor), and growth factors such as *IGF* (insulin like growth factor).

**Fig. 4:**
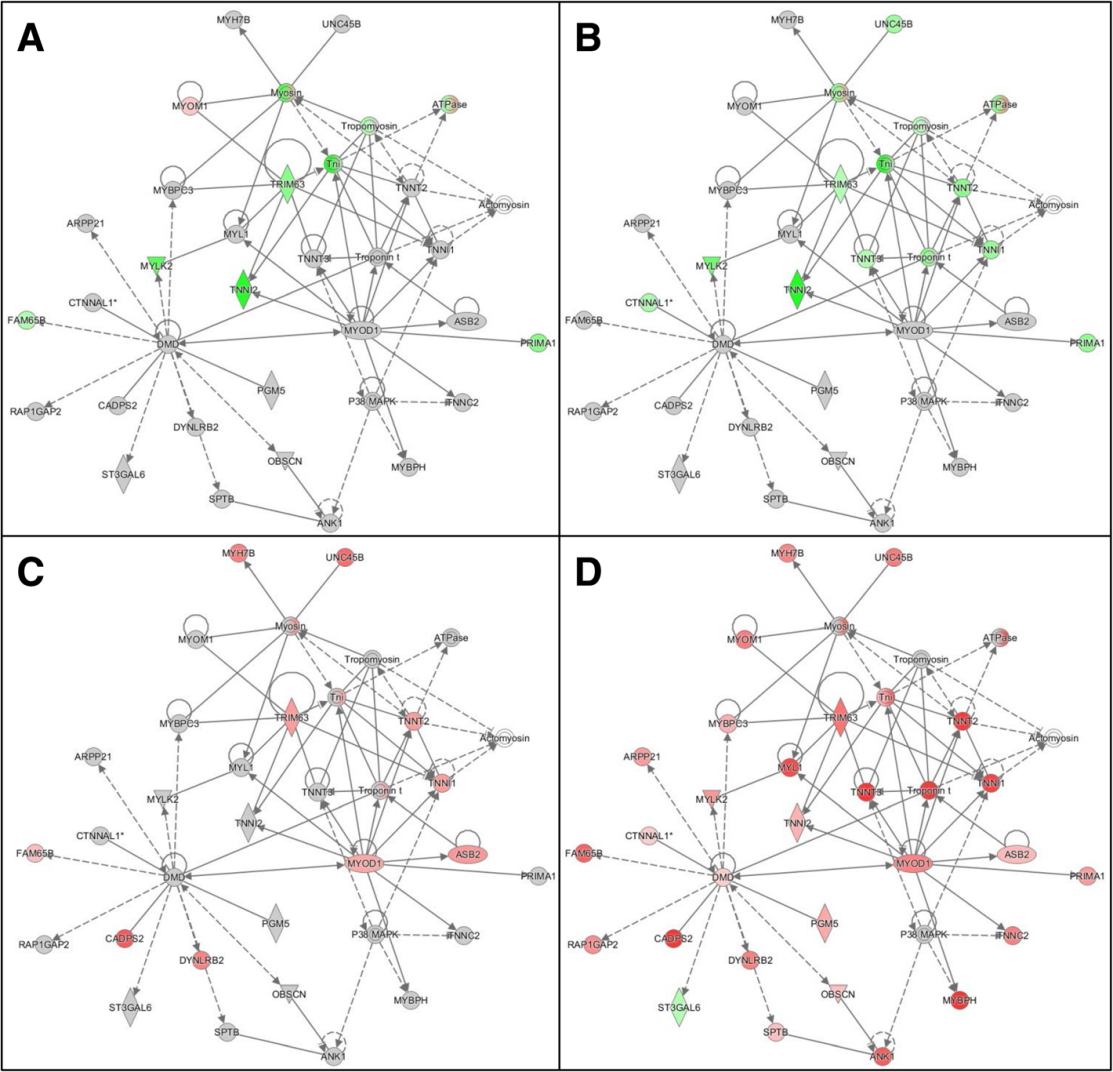
Example gene network identified from differentially expressed genes by Ingenuity Pathway Analysis (IPA) showing differential response of p. major satellite cell transcriptome to temperature. Depicted is the top scoring skeletal and muscle system development network identified by Comparison Analysis. **a**) RBC2 line, 33 °C versus 38 °C. **b**) F line, 33 °C versus 38 °C. **c**). RBC2 line, 43 °C versus 38 °C. **d**) F line, 43 °C versus 38 °C. In each panel the direction (*red* = up regulated, *green* = down regulated) and magnitude (color intensity) of expression changes are indicate for each of the group comparisons. Gray indicates molecule that were included in the dataset but did not meet the significance cutoff value. Shapes within the networks correspond to genes, gene products, or small molecules and “Double circle” symbols indicate gene complexes or groups

The skeletal and muscle system development network depicted in Fig. 4 demonstrates the differential myogenic response in the turkey satellite cells at 33 °C versus 38 °C with several interacting muscle-associated genes being down regulated (Fig. 4a and b). Included within this network are genes such as *TRIM63* (*RNF28*) and *FAM65B* (*C6orf32*). In humans, the TRIM63 protein is involved in cell cycle regulatory processes of striated muscle cells [25]. FAM65B is expressed both in myogenic and non-myogenic primary human cells and is upregulated during muscle cell differentiation [26]. In contrast, many of these same genes were up regulated in cells proliferated at the higher temperature (43 °C versus 38 °C) (Fig. 4c and d). The magnitude of expression change was greater in the F-line (Fig. 4b and d) that in RBC2 (Fig. 4a and c). This is especially evident for members of the Troponin complex (*TNNT1*, *TNNT2*, and *TNNT3*), but also includes genes such as *UNC45B* (Unc-45 Myosin Chaperone B) which plays a role in sarcomere formation during muscle cell development.

The majority of DE genes were also unique to treatment groups (temperature/line) (Additional file 1: Figure S4). However, 14 significant DE genes were shared among all treatment comparisons, identifying a suite of genes that are jointly affected by deviation from the control incubation temperature. These included *CASQ2* (calsequestrin 2) a calcium binding protein that stores calcium for muscle function*, EFEMP1* (EGF containing fibulin-like extracellular matrix protein 1) which may play a role in cell adhesion and migration*,* and genes such as *FAT4* (FAT atypical cadherin 4)*,* and *NKD1* (naked cuticle homolog 1) which help control and maintain planar cell polarity. Others include *IGSF9B* (immunoglobulin superfamily, member 9B)*, NTSR1* (neurotensin receptor 1)*, PTGS2* (prostaglandin-endoperoxide synthase 2)*, RBM46* (RNA binding motif protein 46)*, TPPP3* (tubulin polymerization-promoting protein family member 3) and four model loci (*LOC104910222* [myelin basic protein-like], *LOC104909321* [E-selectin-like], *LOC104911264* [uncharacterized, ncRNA], *LOC104917414* [uncharacterized, ncRNA], and *LOC100541961* [pantetheinase-like]). These 14 genes fall into four categories: up-regulated in both cold and hot treatments relative to control (*EFEMP1*, *IGSF9B*, *LOC104910222*, *NKD1*), down-regulated in both treatments (*NTSR1*, *RBM46*), up-regulated by cold but down-regulated by heat (*FAT4*, *LOC100541961*, *LOC104909321* [selectin L*, SELL*], *LOC104911264*, *LOC104917414*, *PTGS2*) and up-regulated by heat but down-regulated by cold (*CASQ2*, *TPPP3*).

### Effects of selection (Line differences)

**Fig. 5:**
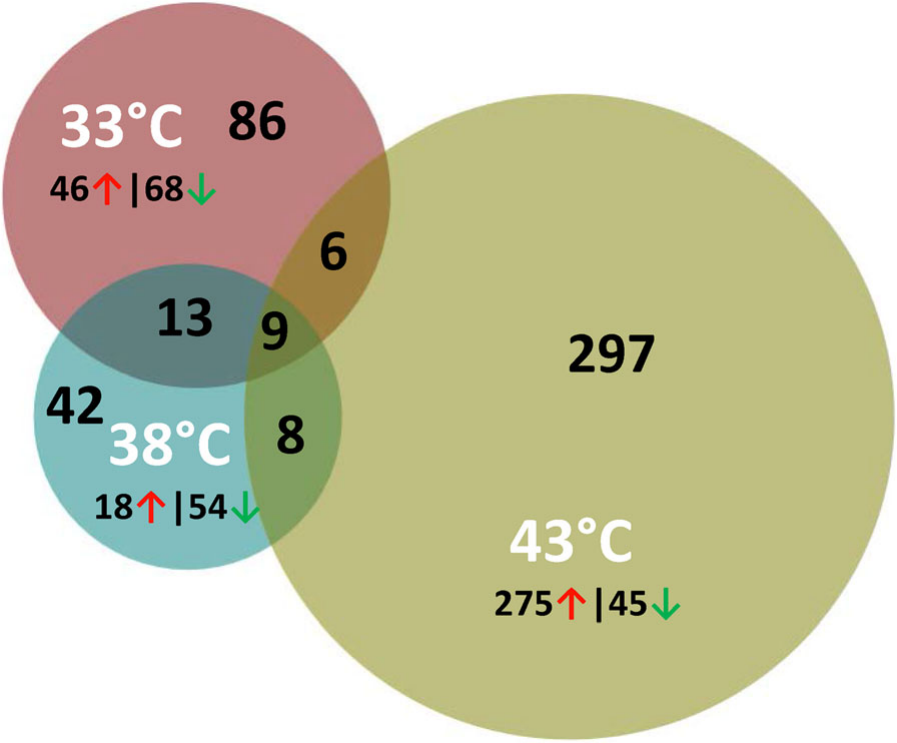
Distribution of differentially expressed genes between lines (F-line versus RBC2) during p. major satellite cell proliferation. For each temperature comparison, the number of genes with FDR pval <0.05 and |Log_2_FC| > 2.0 that were shared or unique to each incubation temperature are indicated. The number and direction of expression change (↑or ↓) for the genes included in each temperature group are listed outside the Venn diagram. Circle size is proportional to the number of genes

At the control temperature 38 °C, 72 genes showed significant DE with |Log_2_FC| > 2.0 in comparisons between the F-line and RBC2 (Additional file 4: Table S3, Additional file 5: Table S4). Of these, 42 were unique to the temperature comparison (Fig. 5). The majority of the unique genes (75%) were down regulated in the F-line. Significantly enriched GO Biological processes involving these 42 genes were synaptic transmission (cell signaling and communication) and system development. Loci unique to 38 °C showing the greatest up regulation in the F-line were LOC104910496 (amphiphysin-like), *DRD1* (dopamine receptor D1), *CAMKK1* (calcium/calmodulin-dependent protein kinase kinase 1, alpha), and those showing the greatest down regulation were *MEOX2* (mesenchyme homeobox 2), *LOC104916656* (uncharacterized, ncRNA), and *SYT1* (synaptotagmin I). Broadly defined these genes function in regulating transcription, neuronal growth and development, exocytosis, and apoptosis.

Greater differences were observed between the genetic lines in the 33 °C and 43 °C thermal challenges. At 33 °C, 114 genes showed significant DE and |Log_2_FC| > 2.0. Of these, 86 were unique to the temperature comparison (Fig. 5). At 43 °C, differential expression of 320 loci was significant (Table 3, Additional file 6: Table S5) with 297 being unique. At 33 °C, 60% of the 86 DE genes were down regulated in the F-line compared to RBC2. Significantly enriched GO Biological processes included nervous system development and cell communication. Loci unique to 33 °C showing the greatest up regulation in the F line were *GAS6* (growth arrest-specific 6) and *LOC100550971* (*P2Y* purinoceptor 1-like), and those showing the greatest down regulation were *KCNIP1* (Kv channel interacting protein 1), *LOC104913529* (uncharacterized, ncRNA), and *LOC104912369* (collagen alpha-1(XI) chain-like) a fibrillar collagen. *GAS6* is a gamma-carboxyglutamic acid (Gla) domain-containing protein thought to be involved in the stimulation of cell proliferation [27]. P2Y purinoceptor 1 belongs to the family of G-protein coupled receptors. Binding of ADP to the P2YR1 receptor is involved in calcium signaling [28]. *KCNIP1* encodes a member of the family of cytosolic voltage-gated potassium (Kv) channel-interacting proteins that regulate neuronal membrane excitability in response to intracellular calcium [29].

**Table 3:** Summary of Wnt family gene expression observed in thermally challenged turkey muscle satellite cells

		33R vs 38R			33F vs 38F			43R vs 38R			43F vs 38F		
Locus	Description		Log_2_FC	FDR pval		Log_2_FC	FDR pval		Log_2_FC	FDR pval		Log_2_FC	FDR pval
LOC104916364	protein Wnt-1	↓	−2.393	NS	↓	−2.339	NS	↓	−2.172	NS	↓	−2.333	NS
LOC104916471	protein Wnt-1-like	-	0.000	NS	↓	−2.339	NS	-	0.000	NS	↓	−2.333	NS
LOC104917060	protein Wnt-1-like	-	0.000	NS	-	0.000	NS	-	0.000	NS	-	0.000	NS
WNT2	wingless-type MMTV integration site family member 2	↑	2.259	NS	-	0.000	NS	↑	2.493	NS	-	0.000	NS
WNT2b	wingless-type MMTV integration site family, member 2B	**↑**	*1.310*	*0.0316*	↓	−1.087	NS	↑	1.163	NS	↓	−0.971	NS
WNT3	wingless-type MMTV integration site family, member 3	-	0.000	NS	↑	2.305	NS	-	0.000	NS	-	0.000	NS
WNT3a	wingless-type MMTV integration site family, member 3A	↑	*5.942*	*0.0003*	↑	*4.237*	*1.04E-05*	-	0.000	NS	↓	−2.336	NS
WNT4	wingless-type MMTV integration site family, member 4	↓	*−0.940*	*0.0000*	↓	*−1.598*	*7.64E-53*	**↓**	−2.552	*6.89E-28*	↓	*−1.880*	*1.3E-57*
WNT5a	wingless-type MMTV integration site family, member 5A	↑	0.007	NS	↑	0.499	NS	↑	0.398	NS	↓	−0.350	NS
WNT5b	wingless-type MMTV integration site family, member 5B	↓	*−0.997*	*1.3E-17*	↓	*−1.388*	*2.4E-28*	↓	*−1.583*	*7.94E-09*	↓	*−1.996*	*1.11E-43*
WNT6	wingless-type MMTV integration site family, member 6	↓	−0.990	NS	↓	3.150	NS	↑	0.885	NS	↑	*5.477*	*0.0109*
WNT7a	wingless-type MMTV integration site family, member 7A	↓	−0.159	NS	↓	−1.806	NS	↑	1.596	NS	↑	*4.528*	*3.93E-19*
WNT7b	wingless-type MMTV integration site family, member 7B	-	0.000	NS	-	0.000	NS	-	0.000	NS	-	0.000	NS
WNT8a	wingless-type MMTV integration site family, member 8A	-	0.000	NS	-	0.000	NS	-	0.000	NS	-	0.000	NS
WNT8b	wingless-type MMTV integration site family, member 8B	↑	0.462	NS	↑	0.472	NS	↓	−1.092	NS	↓	−0.035	NS
WNT9a	wingless-type MMTV integration site family, member 9A	↑	2.382	NS	↑	*5.707*	*0.0013*	↓	−2.172	NS	-	0.000	NS
WNT9b	wingless-type MMTV integration site family, member 9B	-	0.000	NS	-	0.000	NS	-	0.000	NS	-	0.000	NS
WNT10a	wingless-type MMTV integration site family, member 10A	↓	*−0.631*	*0.0033*	↓	−0.218	NS	↑	0.166	NS	↑	0.065	NS
WNT11	wingless-type MMTV integration site family, member 11	-	0.000	NS	-	0.000	NS	-	0.000	NS	-	0.000	NS
LOC100538380	protein Wnt-11b-like	-	0.000	NS	-	0.000	NS	-	0.000	NS	↑	3.155	NS
WNT16	wingless-type MMTV integration site family, member 16	↑	2.251	NS	-	0.000	NS	-	0.000	NS	-	0.000	NS

Numbers in italics indicate comparisons with significant expression change

In contrast to the trend observed at 33 °C and 38 °C, at 43 °C the greatest proportion (85.9%) of the 320 DE genes (|Log_2_FC| > 2.0) were up regulated in the F-line vs RBC2. Of these, 297 loci were uniquely significant to this temperature comparison. Significantly enriched GO Biological processes included muscle contraction, mesoderm and system development, proliferation, differentiation and cell signaling. Loci unique to 43 °C showing the greatest up regulation in the F line included *ACE* (angiotensin I converting enzyme), *LOC104914068* (uncharacterized, ncRNA), *LRRC17* (leucine rich repeat containing 17), *HCK* (HCK proto-oncogene, Src family tyrosine kinase), *FGF13* (fibroblast growth factor 13), *DNASE2B* (deoxyribonuclease II beta), *CCDC153* (coiled-coil domain containing 153), and *TXLNB* (taxilin beta). Greatest down regulation was observed for *LOC100550020* (*DACH1*, dachshund homolog 1). Interestingly, in humans *DACH1* encodes a chromatin-associated transcription factor that acts to regulate cell fate determination during development [30].

Expression differences between the RBC2 and F line are further exemplified by the genes that are shared among the temperature treatments (Fig. 5, Additional file 10: Table S9). For example, 9 genes showed significant (*p*-value < 0.05, and |Log_2_FC| > 2.0) differential expression between the lines at all three temperatures. Interestingly, all nine were down regulated in the F line. Genes showing the greatest expression differences included *TECRL* (trans-2,3-enoyl-CoA reductase-like) and *LOC104915513* (histone deacetylase 7-like) (Additional file 10: Table S9). Although little is known about the function of *TECRL*, this protein with oxidoreductase activity is thought to be involved in the fatty acid biosynthesis and through the pathway *Regulation of lipid metabolism by Peroxisome proliferator-activated receptor alpha (PPARα)* (www.genecards.org). Histone deacetylase 7 is a member of the histone deacetylase family which acts in repression of gene transcription by affecting transcription factor access to DNA [31]. *CTNND2* (catenin (cadherin-associated protein), delta 2) and *GABRA2* (gamma-aminobutyric acid (GABA) A receptor, alpha 2) showed decreased expression with temperature elevation. Both of these genes are described in humans as being active in the mammalian brain. CTNND2 plays a role in cell adhesion and movement [32] and GABRA2 is associated with neurotransmitter inhibition but GO annotations include chloride channel activity.

Comparison of the DE genes shared among the temperature pairs (Fig. 5) found more genes shared between the 33 °C and 38 °C treatments (13 vs 8 and 6) and a similar trend for reduced expression (down regulation) in the F-line (Additional file 10: Table S9). Greatest change was observed for *LOC104916312* (forkhead box protein C2-like) a FOX-family transcription factor with average Log_2_FC = -6.23 (lower) in F versus RBC2 cells. Genes shared between 38 °C and 43 °C showed a mixture of up and down regulation between the lines and genes shared by the two temperature extremes (33 °C and 43 °C) were either mixed in response or upregulated in the F-line. An example of the former is *LOC100541022* (keratin, type I cytoskeletal 19-like) where the gene was down regulated in F-line cells at 33 °C (Log_2_FC = -5.4) but up regulated (2.12) at 43 °C.

## Discussion

Extreme temperature changes are of particular concern to the poultry industry because of the detrimental effects on muscle that ultimately impact meat quality. Satellite cells are multipotential-stem cells located between the basement membrane and sarcolemma of myofibers [10]. As the only source of posthatch myonuclei, these cells are a self-renewing stem cell population responsible for all posthatch skeletal muscle growth [33]. During postnatal development, satellite cells provide myonuclei to promote skeletal muscle growth [34]. In adults, myonuclei promote homeostasis through repair, regeneration or hypertrophy. In poultry, satellite cells are most active the first week after hatch [11, 35].

The function and fate of satellite cells are influenced by the local microenvironment and expression of myogenic regulatory factors [36, 37]. Satellite cells are characterized by their expression of transcription factors (*Pax3* and *Pax7*) and through signaling, activate myogenic determination genes such as *Myf5* and *MyoD* [38]. Quiescent cells are Pax7+ but do not express *MyoD* or myogenin and most are *Myf5* positive [39]. Both *Myf5* and *MyoD* are basic helix-loop-helix transcription factors required for muscle differentiation. Alternate expression of these proteins following proliferation influences cell fate [36]. Proliferating and differentiating myoblasts express *MyoD* whereas myogenin expression occurs during differentiation.

As demonstrated in the thermal challenge of cultured turkey satellite cells, cold treatment resulted in an overrepresentation of genes involved in cell signaling/signal transduction and cell communication (Additional file 7: Table S6). In contrast, heat-treated cells showed greater tendency towards muscle system development and differentiation (Additional file 8: Table S7). Satellite cell proliferation and differentiation is directly influenced by the cell niche including signaling molecules, hormones and innervation [37]. The activation and proliferation of satellite cells is modulated by signaling molecules (*Wnt*, *Notch*, *Myf5*) and growth factors *(IGF*, *FGF*). Satellite cells derived from single muscle fibers quickly reenter the cell cycle, express *MyoD*, and given the necessary substrates, establish proliferative colonies and differentiate into myotubes [39, 40]. In the chicken, proliferating satellite cells express *Pax7* and *MyoD* but not myogenin [41]. *In vivo*, signaling between vascular and satellite cells is required for satellite cell activation and extrinsic signaling molecules orchestrate myogenisis [42]. In the present study, *MYOD1 (MyoD)* and several interacting genes were significantly upregulated in the heat exposed F-line cells (Fig. 4, Additional file 5: Table S4).

Myogenesis is controlled by signaling pathways that direct expression of the chief myogenic regulators. Among these pathways are those activated by Wnt ligands [43]. Wnt ligands are critical signaling molecules controlling various aspects of both muscle development and regeneration. In humans, 19 *Wnt* genes have been described [44]. Chicken genome contains 18 *Wnt* genes that are homologous to the corresponding human and mouse genes [45]. The turkey gene set includes 21 *Wnt* loci of which three correspond to Wnt1 (Table 3). The only mammalian homolog missing in these galliformes is *Wnt10b.* In the chicken, *Wnt3a* and Wnt*7b* encode alternative first exon isoforms. Although the interplay among Wnt pathways is still an area of investigation, it is clear that signaling through both the canonical Wnt/β-catenin and non-canonical Wnt pathways (*PCP* and *mTOR*) are important in myogenesis and neuromuscular synaptogenesis. The Wnt/Ca + signaling pathway was among the top canonical pathways altered in the proliferating turkey satellite cells (Additional file 9: Table S8).

Differentiation of mammalian adult satellite cells is controlled via Wnt signaling which influences the expression of myogenic regulatory factors. *Wnt1*, *3a* and *5a* induce proliferation whereas 4 and 6 are inhibitory [46]. Canonical Wnt signaling and activation of B-catenin/TCF transcriptional complexes regulate stem cell differentiation. Non-canonical Wnt-signaling mediates satellite cell renewal and myofiber growth through activation of pathways such as the AKT/mTOR pathway [42]. Activation of Wnt B-catenin signaling may also be important in modulating early regenerative processes following injury [43]. Several members of the Wnt family are up-regulated in myoblasts and myofibers of regenerating muscle [47]. During regeneration, *Wnt5a*, *5b* and *7a* are upregulated early while *Wnt4* expression is down regulated. *Wnt7b* and *3a* are expressed later. *Wnt7a* is important in the induction of satellite cells through PCP signaling and overexpression of *Wnt7a* results in higher satellite cell numbers. Overexpression of *Wnt4* in chicken embryos enhanced differentiation and increased muscle mass [42].

Significant DE was observed for several of the turkey *Wnt* genes in the challenged satellite cells. *Wnt4* and *5b* were significantly down regulated at both challenge temperatures compared to control and in both lines and were down regulated in all comparisons. *Wnt2b*, *3a*, *9a* and *10a* were significantly up-regulated in cold-treated cells whereas *Wnt6* and *7a* were significantly up-regulated in heat treated satellite cells (Table 3). Some of these genes had only minor expression changes; however, *Wnt3a*, *6*, *7b* and *9a* had Log_2_FC > 4. Only one *Wnt* gene (*Wnt7a* which is linked to the AKT/mTOR pathway is important in the induction of satellite cells) showed significant DE between the RBC2 ad F-lines. At 43 °C, expression of *Wnt7a* was significantly higher in the F-line (Log_2_FC = 3.925, Additional file 5: Table S4). These findings support the importance of cell signaling during proliferation of these satellite cells and suggest targets for further research on the differential control of satellite cell proliferation.

Studies of the self-renewal processes in muscle suggest that the satellite cell pool is heterogeneous; comprised of self-renewing stem cells and predestined myogenic precursors [48]. Myogenic and adipogenic cells may represent separate satellite cell populations. Several studies have reported the non-myogenic differentiation of satellite cells [48]. The capacity for non-myogenic differentiation may ultimately lie in the heterogeneous nature of the satellite cell population [36]. Most satellite cells from myofibers cannot spontaneously differentiate into adipocytes [49]. However, they can be activated in culture to induce osteogenic and apidogenic pathways in addition to myogenesis. Osteogenic differentiation is reportedly induced via bone morphogenetic protein 2 (*BMP2*) and adipogenic fate can be altered by Wnt signaling. Powell et al. [13] found p. major satellite cells to express apidogenic genes when subjected to nutrient restriction. Gene expression of cultured quiescent and regenerating myoblasts may differ significantly from *in vivo* satellite cells [50] as satellite cell progeny in culture are distinct from cells newly isolated from muscle [51, 52].

There is debate regarding the ability of satellite cells to adopt a true osteogenic or apidogenic fate. For example, Starkey et al. [49] suggest that satellite cells accumulate lipid in culture but do not undergo apidogenic differentiation to adopt a new fate even under apidogenic-inducing conditions. *In vivo* results demonstrated both adipocyte formation in the connective tissue surrounding the muscle fiber bundles and within muscle fiber bundles [53, 54]. As interpreted by Smith and Johnson [55], adipocytes formed in the connective tissue between muscle bundles in poultry is similar to true marbling. Deposition of adipocytes within muscle bundles in the avian p. major muscle is consistent with the conversion of muscle satellite cells to adipocytes.

Signaling is important in determining cell fate as myocytes, apidocytes and osteocytes originate from the same embryonic precursor cells. Wnt and Notch signaling controls satellite cell fate. Myoblasts from cultured myofibers only respond to Wnt signaling late in differentiation, whereas Notch signaling regulates proliferation, differentiation and determination of cell fate. Low activity of Wnt signaling early in proliferation may allow for sufficient myoblast expansion via Notch signaling [56]. Commitment to the myocyte lineage may require Wnt signaling as disruption *in vitro* causes transdifferentiation of myoblasts into adipocytes.

Temperature can alter satellite cell proliferation, differentiation and ultimately cell fate [57–60], which can impact skeletal muscle growth and meat quality [58, 61]. Immature poults have an inefficient thermoregulatory system and are sensitive to extreme temperatures. Increased temperatures can affect lipid accumulation and adipogenic gene expression of satellite cells [59]. The increase in intramuscular fat accumulation may be mediated, at least in part, by satellite cells that transdifferentiate to an adipogenic lineage [12, 37, 62, 63]. In mammals, key adipogenic genes are essential for the transcriptional regulation of adipogenesis and is controlled by a cascade of transcription factors led by the CCAAT/enhancer-binding protein (*CEBP*) *β* [64].

Very early in adipocyte differentiation, induction of *CEBPβ* (*CEBPB*) results in the downstream expression of *PPARG* (*PPARγ*) and *CEBPA* (*CEBPα*) as *CEBPB* directly binds to their promoters [65]. Delayed transactivation of *PPARG* and *CEBPA* by *CEBPB* appears necessary for mitotic clonal expansion and progression of terminal differentiation, a process required for adipogenesis [66]. Preadipocytes are maintained in an undifferentiated state through inhibition of CEBPB and PPARG by signaling though Wnt1. However, decrease in Wnt10b may be required for adipogenesis and this ligand may be an endogenous regulator [67]. Interestingly, *CEBPB* also suppresses canonical Wnt-β catenin signaling through transcriptional inhibition of the Wnt ligand (*Wnt10b*) that inhibits apidogenesis [68]. As the *Wnt10b* homolog is absent from the turkey genome, it is not known which of the Wnt family members, or if any directly inhibit apidogenesis.

Expression studies of adipogenic genes in poultry have resulted in mixed outcomes. Harding et al. [59] found satellite cells isolated from the p. major muscle of chickens to have a greater tendency to express adipogenic genes than those isolated from the biceps femoris muscle. Within the p. major satellite cells, *CEBPB* expression, as measured by qRT-PCR, increased with increasing temperature, while *PPARG* expression decreased [59]. In the same satellite cell culture system used in the present study, we [69] found expression of *CEBPB*, *PPARG* and stearoyl-CoA desaturase (*SCD*, an enzyme responsible for complex lipid production), to decrease as temperature increased from 33 °C to 43 °C at 72 h of proliferation despite an observed increase in adiposity.

In the present study significant differences in *CEBPB*, *PPARG* and *SCD* were observed among the temperature/group comparisons, but the degree of expression change in general was small (Log_2_FC < 1.0). For example, *CEBPB* expression significantly decreased in satellite cells from both the RBC2 and F-lines as temperature increased. Expression of *CEBPB* was significantly higher at 33 °C compared to 38 °C in cells from both lines but with low overall fold change (average Log_2_FC = 0.451). Overall expression was slightly lower in F-line cells, but this difference was not significant at any temperature. Expression of *PPARG* was significantly higher at 33 °C compared to 38 °C in cells from both lines but with low overall fold change (average Log_2_FC = 0.897). Expression of *PPARG* at 43 °C was significantly higher in RBC2 cells but significantly lower in F-line cells compared to cells incubated at 38 °C. Again in both comparisons the degree of change was small (Log_2_FC = 1.16 and −0.62, respectively). As nuclear factors, small changes in genes like CEBPB and PPARG may have amplified downstream effects. Expression of *SCD* was higher in F-line cells at all temperatures and this difference was significant at 33 °C and 38 °C. The greatest difference occurred at 33 °C where expression in F-line cells was 1.875 x higher. Expression of SCD was lowest in both lines at 38 °C but increased in both lines at 43 °C (Log_2_FC = 1.32 and 0.772, respectively). These RNAseq results are consistent with our experiments [69] that measured expression of these genes by qRT-PCR in the same satellite cell culture system.

As demonstrated in the present study, temperature significantly alters gene expression in turkey skeletal muscle satellite cells. Numerous significant gene expression differences were observed between cells when incubated at higher (43 °C) or lower (33 °C) temperatures. It is also apparent that genetic selection for 16weeks body weight (muscle mass) has altered satellite cells gene expression. One hypothesis is that selection has altered the satellite cell niche through reduced innervation or limited vascularization. Satellite cells isolated from F line turkeys have increased proliferation and differentiation [15]. Clark et al. [60] found satellite cells of the F line to be more sensitive to temperature changes during proliferation and differentiation. When incubated at temperatures above 38 °C, F line satellite cells also had less intracellular lipid accumulation compared to RBC2 cells. This suggested that growth selection has changed the proportion of resident satellite cells able to convert to an adipogenic lineage in response to thermal cues. Although the ability of the cultured cells to adopt a true non-myogenic fate is unknown, a decrease in adipocyte-like properties could limit *in vivo* transdifferentiation of satellite cells to an adipogenic lineage [70].

Exposure of poultry to extreme temperatures, especially in the critical post-hatch time-frame, can seriously compromise the quality of meat. With climate change, the frequency and intensity of temperature extremes are expected to grow, thereby exacerbating problems with meat production. Worldwide demand for lean, high-quality animal protein continues to expand, and the industry must develop birds that can withstand thermal variation while yielding high-quality meat. Understanding the impact of thermal stress on satellite cells is critical to this effort. The results of this study provide important insights into the proliferation of turkey stem cells in response to thermal challenge.

## Conclusions

This study demonstrates that temperature significantly alters gene expression in satellite cells of turkey skeletal muscle. Numerous gene expression differences were observed between cells incubated at both lower (33 °C) and higher (43 °C) temperatures as compared to control (38 °C). Enrichment analysis indicated a shift at 33 °C towards cell signaling whereas at 43 °C cells had gene expression profiles with a shift towards muscle development. For example, markers of cell proliferation such as *MYOD1* and several interacting genes were significantly upregulated in the heat exposed cells. Cell signaling is critical during satellite cell proliferation and differential expression of chief myogenic regulators and pathways activated by Wnt ligands were observed. The Wnt/Ca + signaling pathway was among the canonical pathways significantly altered in the proliferating turkey satellite cells. Transcriptome analysis found greater differences in gene expression for satellite cells from the growth selected F-line as compared to its random bred control. Genes significantly altered by cold treatment tended to be down regulated in the F-line cells whereas genes significantly altered by heat treatment tended to be up regulated. This study provides important insights into the proliferation of turkey skeletal muscle stem cells in response to thermal challenge and identifies new targets for further research on the differential control of satellite cell proliferation in poultry.

## Additional files

Additional file 1: Figure S1. Box plot of normalized gene expression values for each of the 12 RNAseq libraries. Boxes denote upper and lower quartile with medians displayed as lines within the boxes. **Figure S2**. Hierarchical clustering of samples based on Euclidean distance reiterated relationships shown by PCA. The heat map below is based on experiment-wide normalized gene expression across all groups. **Figure S3**. Volcano plot showing the relationship between the ANOVA p-values and experiment-wise Log_2_ fold change for gene expression in p. major satellite cell transcriptomes during proliferation. **Figure S4**. Distribution of differentially expressed genes for cold (33 °C versus 38 °C) and hot (43 °C versus 38 °C) comparisons of each line (RBC2 and F) during p. major satellite cell proliferation. For each temperature comparison, the number of genes with FDR pval <0.05 and |Log_2_FC| > 2.0 that were shared or unique to each incubation temperature are indicated. (ZIP 2820 kb)

Additional file 2: Table S1. Mean quality-trimmed RNAseq read counts for turkey p. major muscle satellite cells from two lines (RBC2 and F) after 72 h proliferation. Cells were cultured at 33°, 38° or 43 °C. (XLSX 1807 kb)

Additional file 3: Table S2. Normalized mean RNAseq read counts observed in p. major satellite cells from RBC2 and F line turkeys after 72 h proliferation when cultured at 38 °C. Genes are sorted in descending order by average number of reads. (XLSX 1448 kb)

Additional file 4: Table S3. 20 most significant canonical pathways expressed in satellite cell cultures from each line after 72 h of proliferation at 38 °C. (DOCX 15 kb)

Additional file 5: Table S4. Summary of pairwise differential gene expression (DESeq) analysis of p. major satellite cell transcriptomes. Comparisons highlighted in blue have significant FDR p-values (<0.05) and |Log_2_FC| > 2.0. Comparisons highlighted in brown have significant FDR p-values (<0.05) but with |Log_2_FC| < 2.0. (XLSX 4149 kb)

Additional file 6: Table S5. 50 genes showing the greatest differential expression in each pairwise comparison of treatment groups. Genes highlighted red are up-regulated in the comparison whereas genes highlighted in green are down-regulated. (XLSX 34 kb)

Additional file 7: Table S6. Summary of PANTHER Overrepresentation Test of differentially expressed genes in p. major satellite cell cultures after 72 h of proliferation at 33 °C versus 38 °C. DE turkey genes were matched to the chicken gene reference list for analysis in PANTHER. For each annotated Gene Ontology category, the number of genes in the reference list and those differentially expressed in the turkey are given. Fold enrichment is the number of DE genes divided by Expected. P-values are as determined by the binomial statistic. (DOCX 16 kb)

Additional file 8: Table S7. Summary of PANTHER Overrepresentation Test of differentially expressed genes in p. major satellite cell cultures after 72 h of proliferation at 43 °C versus 38 °C. DE turkey genes were matched to the chicken gene reference list for analysis in PANTHER. For each annotated Gene Ontology category, the number of genes in the reference list and those differentially expressed in the turkey are given. Fold enrichment is the number of DE genes divided by Expected. P-values are as determined by the binomial statistic. (DOCX 22 kb)

Additional file 9: Table S8. 10 most significant canonical pathways identified in IPA comparison analysis of DE genes. Included for each temperature comparison are the p-value, ratio and z-score for the RBC2 and F-line comparisons. (XLS 34 kb)

Additional file 10: Table S9. Significant DE genes among comparisons between genetic lines. Genes in each category correspond to the numbers presented in the Venn diagram of Fig. 5. At each temperature the p-val and fold change are given. Genes highlighted in red were up regulated in the F line compared to the RBC2 in all significant comparisons, whereas those highlighted in green were down regulated. Genes highlighted in blue were upregulated in the F-line at one temperature and down regulated at another. (XLSX 15 kb)

## Abbreviations

ANOVA: Analysis of variance
bp: Base pair
CLCGWB: CLC Genomics workbench
DE: Differentially expressed
F: F-line
FC: Fold change
FDR: False discovery rate
GO: Gene ontology
IPA: Ingenuity pathway analysis
ncRNA: Non-coding RNA
PANTHER: Protein analysis through evolutionary relationships
PCA: Principal component analysis
qRT-PCR: Quantitative real time polymerase chain reaction
RBC2: Randombred control 2
RIN: RNA integrity number
RNASeq: RNA sequencing
SRA: Short read archive
tRNA: Transfer RNA
WNT: Wingless/Integrated
