# Supplementary figures and images for: Response of turkey muscle satellite cells to thermal challenge. I. transcriptome effects in proliferating cells
Source: BMC Genomics. 2017 May 6;18:352. doi: 10.1186/s12864-017-3740-4 (PMC5420122; doi:10.1186/s12864-017-3740-4)

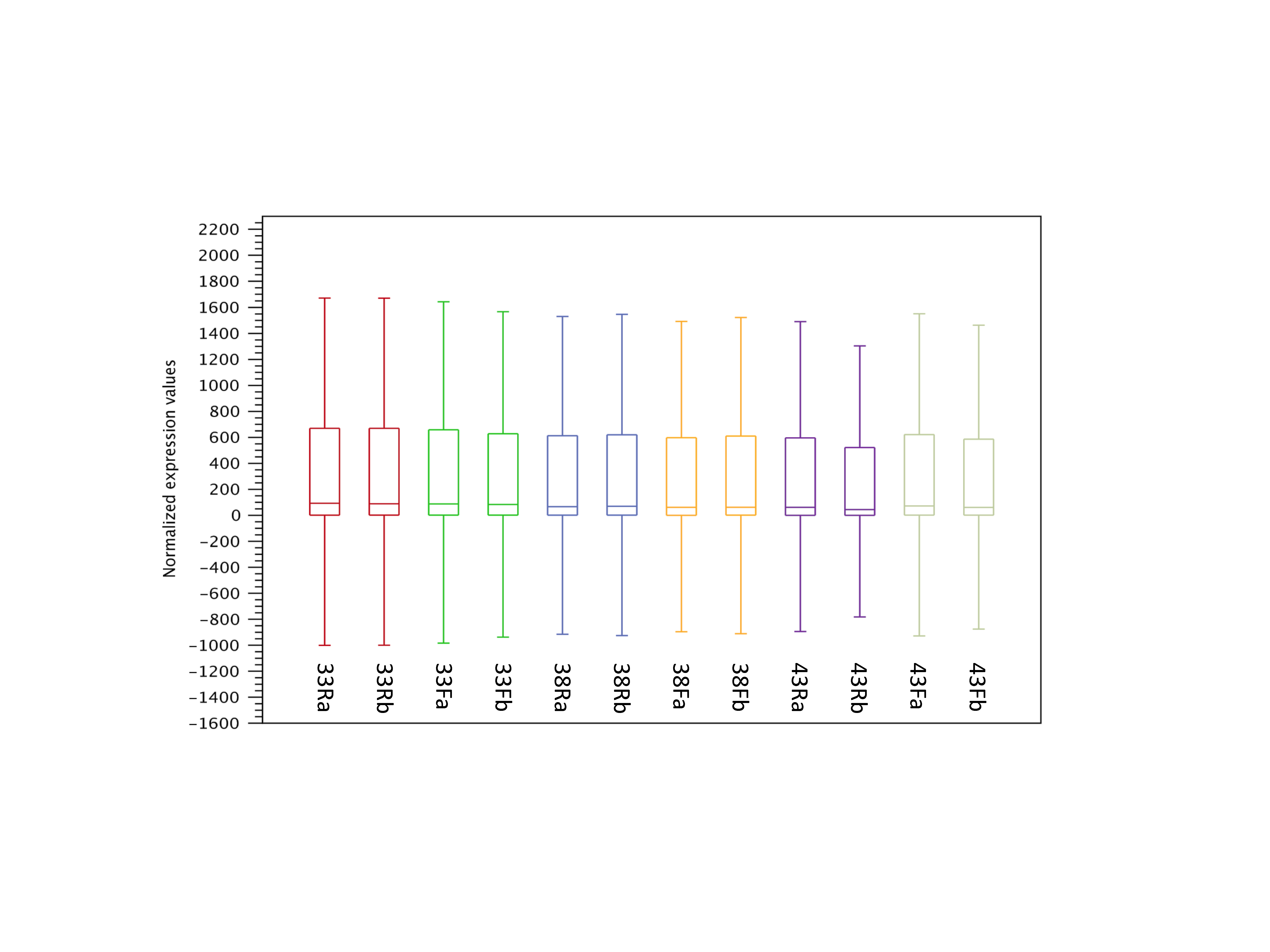

Supplement: Supplementary file 1 — Box plot of normalized gene expression values for each of the 12 RNAseq libraries. Boxes denote upper and lower quartile with medians displayed as lines within the boxes. Figure S2. Hierarchical clustering of samples based on Euclidean distance reiterated relationships shown by PCA. The heat map below is based on experiment-wide normalized gene expression across all groups. Figure S3. Volcano plot showing the relationship between the ANOVA p-values and experiment-wise Log2 fold change for gene expression in p. major satellite cell transcriptomes during proliferation. Figure S4. Distribution of differentially expressed genes for cold (33 °C versus 38 °C) and hot (43 °C versus 38 °C) comparisons of each line (RBC2 and F) during p. major satellite cell proliferation. For each temperature comparison, the number of genes with FDR pval <0.05 and |Log2FC| > 2.0 that were shared or unique to each incubation temperature are indicated. (ZIP 2820 kb) [file 12864_2017_3740_MOESM1_ESM.zip › FigS1.tiff]

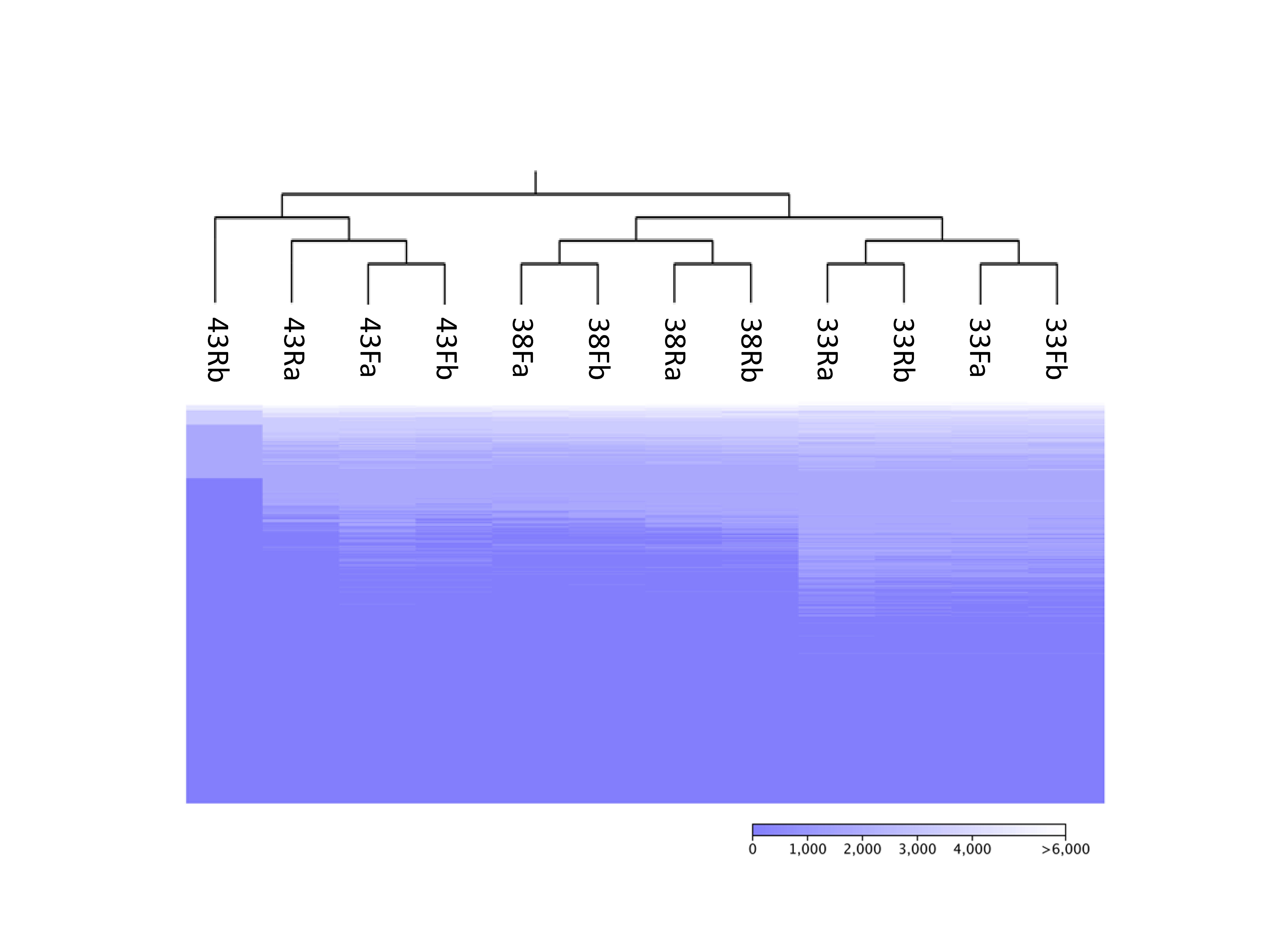

Supplement: Supplementary file 1 — Box plot of normalized gene expression values for each of the 12 RNAseq libraries. Boxes denote upper and lower quartile with medians displayed as lines within the boxes. Figure S2. Hierarchical clustering of samples based on Euclidean distance reiterated relationships shown by PCA. The heat map below is based on experiment-wide normalized gene expression across all groups. Figure S3. Volcano plot showing the relationship between the ANOVA p-values and experiment-wise Log2 fold change for gene expression in p. major satellite cell transcriptomes during proliferation. Figure S4. Distribution of differentially expressed genes for cold (33 °C versus 38 °C) and hot (43 °C versus 38 °C) comparisons of each line (RBC2 and F) during p. major satellite cell proliferation. For each temperature comparison, the number of genes with FDR pval <0.05 and |Log2FC| > 2.0 that were shared or unique to each incubation temperature are indicated. (ZIP 2820 kb) [file 12864_2017_3740_MOESM1_ESM.zip › FigS2.tiff]

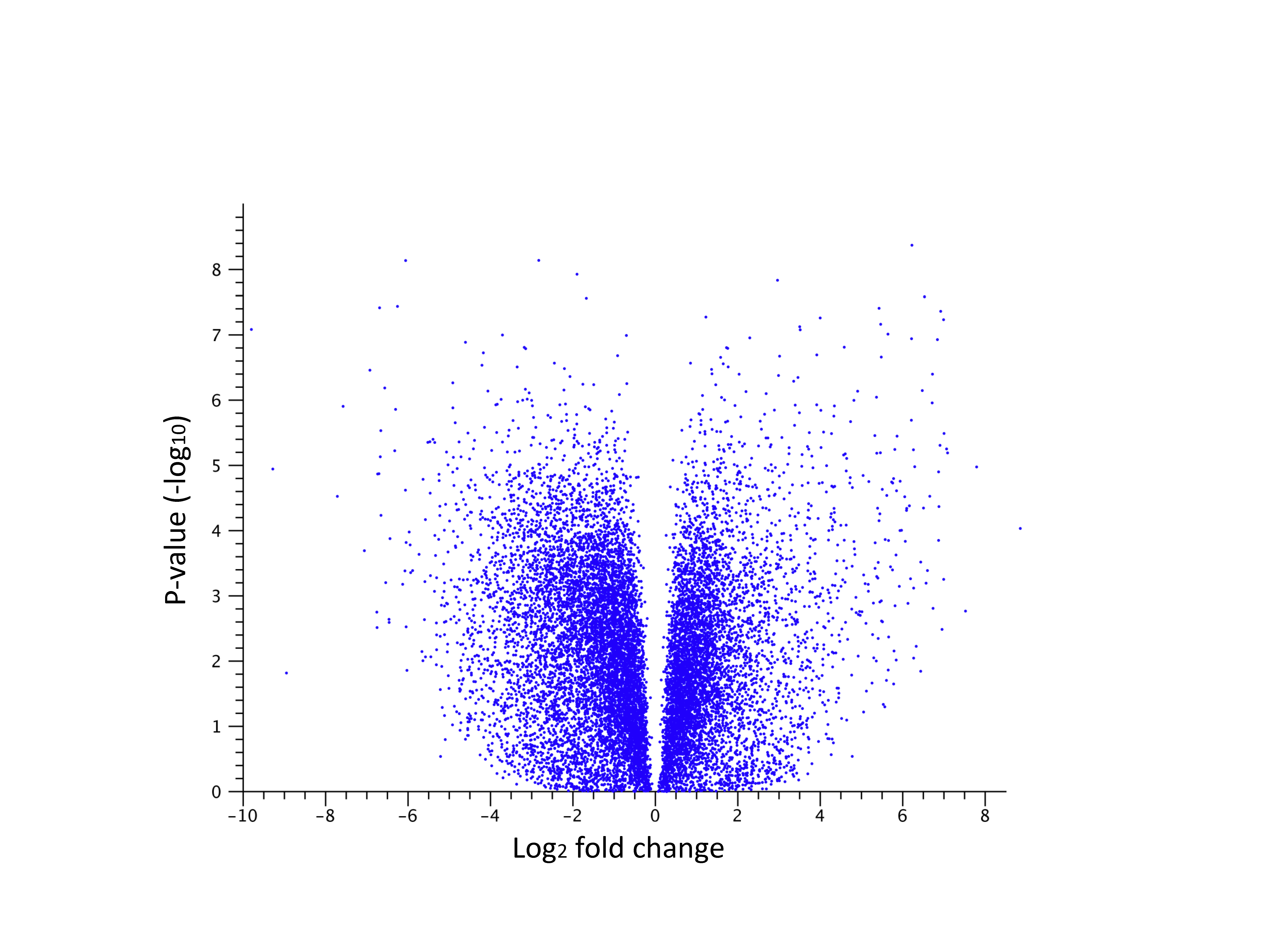

Supplement: Supplementary file 1 — Box plot of normalized gene expression values for each of the 12 RNAseq libraries. Boxes denote upper and lower quartile with medians displayed as lines within the boxes. Figure S2. Hierarchical clustering of samples based on Euclidean distance reiterated relationships shown by PCA. The heat map below is based on experiment-wide normalized gene expression across all groups. Figure S3. Volcano plot showing the relationship between the ANOVA p-values and experiment-wise Log2 fold change for gene expression in p. major satellite cell transcriptomes during proliferation. Figure S4. Distribution of differentially expressed genes for cold (33 °C versus 38 °C) and hot (43 °C versus 38 °C) comparisons of each line (RBC2 and F) during p. major satellite cell proliferation. For each temperature comparison, the number of genes with FDR pval <0.05 and |Log2FC| > 2.0 that were shared or unique to each incubation temperature are indicated. (ZIP 2820 kb) [file 12864_2017_3740_MOESM1_ESM.zip › FigS3.tiff]

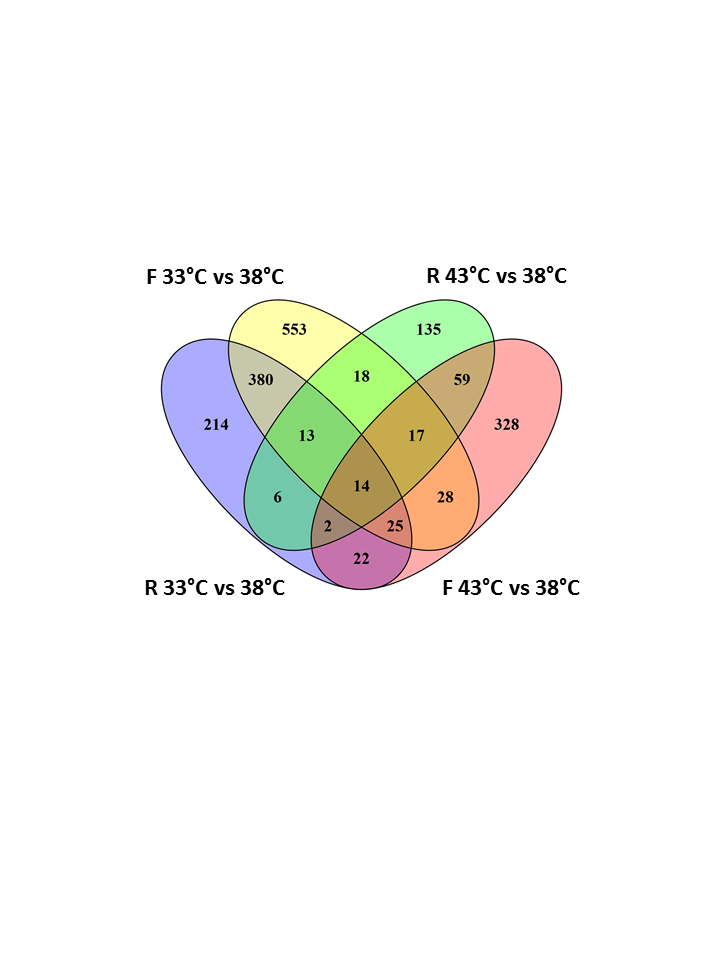

Supplement: Supplementary file 1 — Box plot of normalized gene expression values for each of the 12 RNAseq libraries. Boxes denote upper and lower quartile with medians displayed as lines within the boxes. Figure S2. Hierarchical clustering of samples based on Euclidean distance reiterated relationships shown by PCA. The heat map below is based on experiment-wide normalized gene expression across all groups. Figure S3. Volcano plot showing the relationship between the ANOVA p-values and experiment-wise Log2 fold change for gene expression in p. major satellite cell transcriptomes during proliferation. Figure S4. Distribution of differentially expressed genes for cold (33 °C versus 38 °C) and hot (43 °C versus 38 °C) comparisons of each line (RBC2 and F) during p. major satellite cell proliferation. For each temperature comparison, the number of genes with FDR pval <0.05 and |Log2FC| > 2.0 that were shared or unique to each incubation temperature are indicated. (ZIP 2820 kb) [file 12864_2017_3740_MOESM1_ESM.zip › FigS4.tif]
