# Supplementary material for: Response of turkey muscle satellite cells to thermal challenge. I. transcriptome effects in proliferating cells
Source: BMC Genomics. 2017 May 6;18:352. doi: 10.1186/s12864-017-3740-4 (PMC5420122; doi:10.1186/s12864-017-3740-4)
Supplement: Supplementary file 4 — 20 most significant canonical pathways expressed in satellite cell cultures from each line after 72 h of proliferation at 38 °C. (DOCX 15 kb) [file 12864_2017_3740_MOESM4_ESM.docx]

**Table S3**. **20 most significant canonical pathways expressed in satellite cell cultures from each line after 72 hr of proliferation at 38° C.**

|  | **RBC2** | | | **F-Line** | |  |
| --- | --- | --- | --- | --- | --- | --- |
| **Metabolic Pathways** | **-log(p-value)** | **Ratio** | **-log(p-value)** | | **Ratio** | |
| tRNA Charging | 8.890 | 0.846 | 9.000 | | 0.846 | |
| Superpathway of Inositol Phosphate Compounds | 7.910 | 0.570 | 7.400 | | 0.560 | |
| 3-phosphoinositide Biosynthesis | 6.930 | 0.577 | 6.730 | | 0.571 | |
| 3-phosphoinositide Degradation | 6.440 | 0.575 | 5.880 | | 0.562 | |
| Colanic Acid Building Blocks Biosynthesis | 6.040 | 1.000 | 6.090 | | 1.000 | |
| D-myo-inositol (1,4,5,6)-Tetrakisphosphate Biosynthesis | 5.330 | 0.566 | 5.110 | | 0.558 | |
| D-myo-inositol (3,4,5,6)-tetrakisphosphate Biosynthesis | 5.330 | 0.566 | 5.110 | | 0.558 | |
| D-myo-inositol-5-phosphate Metabolism | 4.550 | 0.538 | 4.090 | | 0.524 | |
| Superpathway of D-myo-inositol (1,4,5)-trisphosphate Metabolism | 4.510 | 0.792 | 4.560 | | 0.792 | |
| TCA Cycle II (Eukaryotic) | 4.180 | 0.783 | 4.230 | | 0.783 | |
| Valine Degradation I | 4.120 | 0.833 | 3.330 | | 0.778 | |
| Superpathway of Cholesterol Biosynthesis | 3.640 | 0.714 | 3.690 | | 0.714 | |
| Pyridoxal 5'-phosphate Salvage Pathway | 3.640 | 0.594 | 3.320 | | 0.578 | |
| Isoleucine Degradation I | 3.580 | 0.857 | 3.620 | | 0.857 | |
| Fatty Acid β-oxidation I | 3.570 | 0.688 | 3.630 | | 0.688 | |
| D-myo-inositol (1,4,5)-trisphosphate Degradation | 3.290 | 0.778 | 3.330 | | 0.778 | |
| 1D-myo-inositol Hexakisphosphate Biosynthesis II (Mammalian) | 2.890 | 0.737 | 2.930 | | 0.737 | |
| D-myo-inositol (1,3,4)-trisphosphate Biosynthesis | 2.890 | 0.737 | 2.930 | | 0.737 | |
| GDP-mannose Biosynthesis | 2.590 | 1.000 | 2.610 | | 1.000 | |
| Salvage Pathways of Pyrimidine Ribonucleotides | 2.550 | 0.516 | 2.350 | | 0.505 | |
|  |  |  |  | |  | |
| **Signaling Pathways** |  |  |  | |  | |
| Protein Ubiquitination Pathway | 26.500 | 0.702 | 26.900 | | 0.702 | |
| EIF2 Signaling | 23.700 | 0.739 | 24.100 | | 0.739 | |
| Regulation of eIF4 and p70S6K Signaling | 18.300 | 0.733 | 18.600 | | 0.733 | |
| NRF2-mediated Oxidative Stress Response | 15.800 | 0.672 | 16.100 | | 0.672 | |
| mTOR Signaling | 15.400 | 0.663 | 15.700 | | 0.663 | |
| Molecular Mechanisms of Cancer | 14.000 | 0.567 | 12.900 | | 0.556 | |
| Estrogen Receptor Signaling | 13.100 | 0.695 | 12.200 | | 0.680 | |
| Hereditary Breast Cancer Signaling | 12.800 | 0.690 | 13.000 | | 0.690 | |
| Role of BRCA1 in DNA Damage Response | 12.100 | 0.769 | 12.300 | | 0.769 | |
| Huntington's Disease Signaling | 10.200 | 0.581 | 10.100 | | 0.576 | |
| PI3K/AKT Signaling | 9.590 | 0.650 | 10.300 | | 0.659 | |
| Aldosterone Signaling in Epithelial Cells | 9.360 | 0.618 | 9.120 | | 0.612 | |
| Adipogenesis pathway | 9.330 | 0.634 | 8.130 | | 0.612 | |
| Chronic Myeloid Leukemia Signaling | 9.330 | 0.688 | 9.490 | | 0.688 | |
| Glucocorticoid Receptor Signaling | 8.990 | 0.549 | 8.300 | | 0.538 | |
| Hypoxia Signaling in the Cardiovascular System | 8.780 | 0.738 | 8.910 | | 0.738 | |
| IGF-1 Signaling | 8.720 | 0.670 | 8.870 | | 0.670 | |
| Death Receptor Signaling | 8.490 | 0.674 | 8.650 | | 0.674 | |
| Integrin Signaling | 8.080 | 0.565 | 8.300 | | 0.565 | |
| HIPPO signaling | 8.010 | 0.674 | 8.150 | | 0.674 | |
