# Supplementary material for: Response of turkey muscle satellite cells to thermal challenge. I. transcriptome effects in proliferating cells
Source: BMC Genomics. 2017 May 6;18:352. doi: 10.1186/s12864-017-3740-4 (PMC5420122; doi:10.1186/s12864-017-3740-4)
Supplement: Supplementary file 7 — Summary of PANTHER Overrepresentation Test of differentially expressed genes in p. major satellite cell cultures after 72 h of proliferation at 33 °C versus 38 °C. DE turkey genes were matched to the chicken gene reference list for analysis in PANTHER. For each annotated Gene Ontology category, the number of genes in the reference list and those differentially expressed in the turkey are given. Fold enrichment is the number of DE genes divided by Expected. P-values are as determined by the binomial statistic. (DOCX 16 kb) [file 12864_2017_3740_MOESM7_ESM.docx]

**Table S6**. **Summary of PANTHER Overrepresentation Test of differentially expressed genes in p. major satellite cell cultures after 72 hr of proliferation at 33° C versus 38° C.** DE turkey genes were matched to the chicken gene reference list for analysis in PANTHER. For each annotated Gene Ontology category, the number of genes in the reference list and those differentially expressed in the turkey are given. Fold enrichment is the number of DE genes divided by Expected. P-values are as determined by the binomial statistic.

| **Biological process** | **Gallus gallus - REFLIST (15696)** | **DE turkey genes (433 of 1292)** | **Expected** | **over / under** | **Fold Enrichment** | **P-value** |
| --- | --- | --- | --- | --- | --- | --- |
| muscle system process (GO:0003012) | 119 | 15 | 3.28 | + | 4.57 | 9.89E-03 |
| synaptic transmission (GO:0007268) | 249 | 23 | 6.87 | + | 3.35 | 4.25E-03 |
| cell-cell signaling (GO:0007267) | 389 | 32 | 10.73 | + | 2.98 | 3.97E-04 |
| single organism signaling (GO:0044700) | 2638 | 132 | 72.77 | + | 1.81 | 9.73E-09 |
| signaling (GO:0023052) | 2641 | 132 | 72.86 | + | 1.81 | 1.06E-08 |
| signal transduction (GO:0007165) | 2440 | 120 | 67.31 | + | 1.78 | 4.86E-07 |
| cell communication (GO:0007154) | 2723 | 132 | 75.12 | + | 1.76 | 9.25E-08 |
| regulation of signaling (GO:0023051) | 1725 | 80 | 47.59 | + | 1.68 | 1.59E-02 |
| regulation of cell communication (GO:0010646) | 1810 | 83 | 49.93 | + | 1.66 | 1.53E-02 |
| positive regulation of biological process (GO:0048518) | 3228 | 147 | 89.05 | + | 1.65 | 3.82E-07 |
| positive regulation of metabolic process (GO:0009893) | 2103 | 95 | 58.01 | + | 1.64 | 4.53E-03 |
| positive regulation of cellular process (GO:0048522) | 2775 | 122 | 76.55 | + | 1.59 | 2.84E-04 |
| multicellular organismal process (GO:0032501) | 3311 | 144 | 91.34 | + | 1.58 | 1.72E-05 |
| single-multicellular organism process (GO:0044707) | 3202 | 139 | 88.33 | + | 1.57 | 4.34E-05 |
| cellular response to stimulus (GO:0051716) | 3223 | 135 | 88.91 | + | 1.52 | 7.22E-04 |
| anatomical structure development (GO:0048856) | 2717 | 113 | 74.95 | + | 1.51 | 1.75E-02 |
| developmental process (GO:0032502) | 2993 | 120 | 82.57 | + | 1.45 | 4.58E-02 |
| response to stimulus (GO:0050896) | 3957 | 158 | 109.16 | + | 1.45 | 7.65E-04 |
| regulation of cellular process (GO:0050794) | 6148 | 242 | 169.6 | + | 1.43 | 8.88E-09 |
| regulation of biological process (GO:0050789) | 6481 | 255 | 178.79 | + | 1.43 | 7.39E-10 |
| biological regulation (GO:0065007) | 6852 | 267 | 189.02 | + | 1.41 | 2.24E-10 |
| single-organism cellular process (GO:0044763) | 6949 | 252 | 191.7 | + | 1.31 | 2.40E-05 |
| single-organism process (GO:0044699) | 8061 | 281 | 222.38 | + | 1.26 | 5.03E-05 |
| cellular process (GO:0009987) | 8784 | 287 | 242.32 | + | 1.18 | 4.38E-02 |
| Unclassified (UNCLASSIFIED) | 4463 | 85 | 123.12 | - | 0.69 | 0.00E+00 |
|  |  |  |  |  |  |  |
| **Cellular component** |  |  |  |  |  |  |
| sarcomere (GO:0030017) | 106 | 12 | 2.92 | + | 4.1 | 4.91E-02 |
| ion channel complex (GO:0034702) | 178 | 17 | 4.91 | + | 3.46 | 1.29E-02 |
| transmembrane transporter complex (GO:1902495) | 196 | 18 | 5.41 | + | 3.33 | 1.21E-02 |
| transporter complex (GO:1990351) | 198 | 18 | 5.46 | + | 3.3 | 1.38E-02 |
| extracellular matrix (GO:0031012) | 293 | 24 | 8.08 | + | 2.97 | 3.14E-03 |
| synapse (GO:0045202) | 352 | 26 | 9.71 | + | 2.68 | 7.59E-03 |
| integral component of plasma membrane (GO:0005887) | 687 | 42 | 18.95 | + | 2.22 | 1.69E-03 |
| intrinsic component of plasma membrane (GO:0031226) | 718 | 42 | 19.81 | + | 2.12 | 4.98E-03 |
| neuron part (GO:0097458) | 605 | 35 | 16.69 | + | 2.1 | 3.89E-02 |
| plasma membrane part (GO:0044459) | 1265 | 65 | 34.9 | + | 1.86 | 1.00E-03 |
| plasma membrane (GO:0005886) | 2327 | 118 | 64.19 | + | 1.84 | 1.79E-08 |
| cell periphery (GO:0071944) | 2400 | 120 | 66.21 | + | 1.81 | 2.79E-08 |
| intrinsic component of membrane (GO:0031224) | 2454 | 101 | 67.7 | + | 1.49 | 1.82E-02 |
| integral component of membrane (GO:0016021) | 2397 | 98 | 66.13 | + | 1.48 | 3.28E-02 |
| membrane (GO:0016020) | 4998 | 181 | 137.88 | + | 1.31 | 8.03E-03 |
| intracellular membrane-bounded organelle (GO:0043231) | 6248 | 132 | 172.36 | - | 0.77 | 3.36E-02 |
| Unclassified (UNCLASSIFIED) | 4455 | 92 | 122.9 | - | 0.75 | 0.00E+00 |
| intracellular organelle part (GO:0044446) | 4341 | 82 | 119.75 | - | 0.68 | 1.59E-02 |
|  |  |  |  |  |  |  |
| **Molecular function** |  |  |  |  |  |  |
| calcium ion binding (GO:0005509) | 467 | 30 | 12.88 | + | 2.33 | 4.12E-02 |
| signal transducer activity (GO:0004871) | 831 | 45 | 22.92 | + | 1.96 | 2.93E-02 |
| Unclassified (UNCLASSIFIED) | 5075 | 104 | 140 | - | 0.74 | 0.00E+00 |
