# Supplementary material for: Response of turkey muscle satellite cells to thermal challenge. I. transcriptome effects in proliferating cells
Source: BMC Genomics. 2017 May 6;18:352. doi: 10.1186/s12864-017-3740-4 (PMC5420122; doi:10.1186/s12864-017-3740-4)
Supplement: Supplementary file 8 — Summary of PANTHER Overrepresentation Test of differentially expressed genes in p. major satellite cell cultures after 72 h of proliferation at 43 °C versus 38 °C. DE turkey genes were matched to the chicken gene reference list for analysis in PANTHER. For each annotated Gene Ontology category, the number of genes in the reference list and those differentially expressed in the turkey are given. Fold enrichment is the number of DE genes divided by Expected. P-values are as determined by the binomial statistic. (DOCX 22 kb) [file 12864_2017_3740_MOESM8_ESM.docx]

**Table S7. Summary of PANTHER Overrepresentation Test of differentially expressed genes in p. major satellite cell cultures after 72 hr of proliferation at 43° C versus 38° C.** DE turkey genes were matched to the chicken gene reference list for analysis in PANTHER. For each annotated Gene Ontology category, the number of genes in the reference list and those differentially expressed in the turkey are given. Fold enrichment is the number of DE genes divided by Expected. P-values are as determined by the binomial statistic.

| **Biological process** | **Gallus gallus - REFLIST (15696)** | **DE turkey genes (307 of 667)** | **Expected** | **over / under** | **Fold Enrichment** | **P-value** |
| --- | --- | --- | --- | --- | --- | --- |
| synaptic transmission, cholinergic (GO:0007271) | 21 | 6 | 0.41 | + | > 5 | 2.60E-02 |
| regulation of myotube differentiation (GO:0010830) | 28 | 7 | 0.55 | + | > 5 | 9.89E-03 |
| regulation of striated muscle cell differentiation (GO:0051153) | 50 | 8 | 0.98 | + | > 5 | 4.70E-02 |
| muscle contraction (GO:0006936) | 92 | 14 | 1.8 | + | > 5 | 3.68E-05 |
| regulation of muscle contraction (GO:0006937) | 80 | 12 | 1.56 | + | > 5 | 5.22E-04 |
| regulation of muscle system process (GO:0090257) | 103 | 14 | 2.01 | + | > 5 | 1.48E-04 |
| muscle system process (GO:0003012) | 119 | 15 | 2.33 | + | > 5 | 1.25E-04 |
| regulation of system process (GO:0044057) | 237 | 19 | 4.64 | + | 4.1 | 1.94E-03 |
| muscle structure development (GO:0061061) | 293 | 23 | 5.73 | + | 4.01 | 1.61E-04 |
| cell-cell signaling (GO:0007267) | 389 | 30 | 7.61 | + | 3.94 | 1.71E-06 |
| synaptic transmission (GO:0007268) | 249 | 19 | 4.87 | + | 3.9 | 4.03E-03 |
| regulation of membrane potential (GO:0042391) | 210 | 16 | 4.11 | + | 3.9 | 3.13E-02 |
| cell adhesion (GO:0007155) | 543 | 30 | 10.62 | + | 2.82 | 2.60E-03 |
| biological adhesion (GO:0022610) | 546 | 30 | 10.68 | + | 2.81 | 2.91E-03 |
| system process (GO:0003008) | 779 | 42 | 15.24 | + | 2.76 | 2.12E-05 |
| circulatory system development (GO:0072359) | 521 | 28 | 10.19 | + | 2.75 | 1.08E-02 |
| cardiovascular system development (GO:0072358) | 521 | 28 | 10.19 | + | 2.75 | 1.08E-02 |
| regulation of cellular localization (GO:0060341) | 653 | 32 | 12.77 | + | 2.51 | 1.35E-02 |
| regulation of transport (GO:0051049) | 957 | 43 | 18.72 | + | 2.3 | 2.21E-03 |
| tissue development (GO:0009888) | 1043 | 44 | 20.4 | + | 2.16 | 8.48E-03 |
| cell development (GO:0048468) | 1012 | 42 | 19.79 | + | 2.12 | 2.26E-02 |
| organ development (GO:0048513) | 1688 | 69 | 33.02 | + | 2.09 | 1.54E-05 |
| regulation of multicellular organismal process (GO:0051239) | 1520 | 62 | 29.73 | + | 2.09 | 1.32E-04 |
| single-multicellular organism process (GO:0044707) | 3202 | 127 | 62.63 | + | 2.03 | 3.35E-13 |
| cell differentiation (GO:0030154) | 1905 | 75 | 37.26 | + | 2.01 | 1.31E-05 |
| system development (GO:0048731) | 2280 | 89 | 44.59 | + | 2 | 3.40E-07 |
| regulation of biological quality (GO:0065008) | 1797 | 70 | 35.15 | + | 1.99 | 8.20E-05 |
| anatomical structure morphogenesis (GO:0009653) | 1389 | 54 | 27.17 | + | 1.99 | 5.67E-03 |
| regulation of localization (GO:0032879) | 1353 | 52 | 26.46 | + | 1.96 | 1.30E-02 |
| multicellular organismal process (GO:0032501) | 3311 | 127 | 64.76 | + | 1.96 | 4.97E-12 |
| multicellular organismal development (GO:0007275) | 2498 | 95 | 48.86 | + | 1.94 | 2.41E-07 |
| anatomical structure development (GO:0048856) | 2717 | 103 | 53.14 | + | 1.94 | 2.76E-08 |
| regulation of developmental process (GO:0050793) | 1351 | 51 | 26.42 | + | 1.93 | 2.74E-02 |
| cellular developmental process (GO:0048869) | 2047 | 76 | 40.04 | + | 1.9 | 1.26E-04 |
| developmental process (GO:0032502) | 2993 | 111 | 58.54 | + | 1.9 | 1.02E-08 |
| single-organism developmental process (GO:0044767) | 2971 | 110 | 58.11 | + | 1.89 | 1.53E-08 |
| cell communication (GO:0007154) | 2723 | 100 | 53.26 | + | 1.88 | 4.27E-07 |
| single organism signaling (GO:0044700) | 2638 | 96 | 51.6 | + | 1.86 | 2.06E-06 |
| signaling (GO:0023052) | 2641 | 96 | 51.66 | + | 1.86 | 2.19E-06 |
| regulation of signaling (GO:0023051) | 1725 | 62 | 33.74 | + | 1.84 | 1.06E-02 |
| regulation of cell communication (GO:0010646) | 1810 | 64 | 35.4 | + | 1.81 | 1.19E-02 |
| signal transduction (GO:0007165) | 2440 | 86 | 47.72 | + | 1.8 | 1.17E-04 |
| regulation of response to stimulus (GO:0048583) | 2005 | 69 | 39.22 | + | 1.76 | 1.11E-02 |
| cellular response to stimulus (GO:0051716) | 3223 | 104 | 63.04 | + | 1.65 | 2.14E-04 |
| positive regulation of biological process (GO:0048518) | 3228 | 103 | 63.14 | + | 1.63 | 4.63E-04 |
| positive regulation of cellular process (GO:0048522) | 2775 | 86 | 54.28 | + | 1.58 | 3.04E-02 |
| response to stimulus (GO:0050896) | 3957 | 122 | 77.4 | + | 1.58 | 9.37E-05 |
| single-organism cellular process (GO:0044763) | 6949 | 198 | 135.92 | + | 1.46 | 4.69E-09 |
| regulation of biological process (GO:0050789) | 6481 | 183 | 126.76 | + | 1.44 | 4.58E-07 |
| biological regulation (GO:0065007) | 6852 | 192 | 134.02 | + | 1.43 | 1.29E-07 |
| single-organism process (GO:0044699) | 8061 | 224 | 157.67 | + | 1.42 | 4.91E-11 |
| regulation of cellular process (GO:0050794) | 6148 | 170 | 120.25 | + | 1.41 | 3.99E-05 |
| cellular process (GO:0009987) | 8784 | 212 | 171.81 | + | 1.23 | 1.02E-02 |
| biological_process (GO:0008150) | 11233 | 269 | 219.71 | + | 1.22 | 7.32E-08 |
| Unclassified (UNCLASSIFIED) | 4463 | 38 | 87.29 | - | 0.44 | 0.00E+00 |
| cellular nitrogen compound metabolic process (GO:0034641) | 2268 | 19 | 44.36 | - | 0.43 | 2.55E-02 |
| heterocycle metabolic process (GO:0046483) | 1962 | 13 | 38.38 | - | 0.34 | 3.56E-03 |
| cellular aromatic compound metabolic process (GO:0006725) | 1973 | 13 | 38.59 | - | 0.34 | 3.02E-03 |
| nucleobase-containing compound metabolic process (GO:0006139) | 1880 | 10 | 36.77 | - | 0.27 | 2.85E-04 |
| nucleic acid metabolic process (GO:0090304) | 1577 | 7 | 30.84 | - | 0.23 | 5.69E-04 |
|  |  |  |  |  |  |  |
| **GO cellular component complete** |  |  |  |  |  |  |
| troponin complex (GO:0005861) | 7 | 4 | 0.14 | + | > 5 | 1.23E-02 |
| myofilament (GO:0036379) | 14 | 6 | 0.27 | + | > 5 | 4.22E-04 |
| striated muscle thin filament (GO:0005865) | 14 | 6 | 0.27 | + | > 5 | 4.22E-04 |
| sarcomere (GO:0030017) | 106 | 22 | 2.07 | + | > 5 | 5.75E-13 |
| sarcoplasmic reticulum (GO:0016529) | 29 | 6 | 0.57 | + | > 5 | 2.61E-02 |
| contractile fiber part (GO:0044449) | 112 | 22 | 2.19 | + | > 5 | 1.74E-12 |
| myofibril (GO:0030016) | 124 | 23 | 2.43 | + | > 5 | 1.30E-12 |
| contractile fiber (GO:0043292) | 131 | 23 | 2.56 | + | > 5 | 4.07E-12 |
| I band (GO:0031674) | 74 | 12 | 1.45 | + | > 5 | 3.76E-05 |
| Z disc (GO:0030018) | 64 | 9 | 1.25 | + | > 5 | 5.92E-03 |
| postsynaptic membrane (GO:0045211) | 81 | 10 | 1.58 | + | > 5 | 5.63E-03 |
| synaptic membrane (GO:0097060) | 94 | 10 | 1.84 | + | > 5 | 2.00E-02 |
| postsynapse (GO:0098794) | 166 | 15 | 3.25 | + | 4.62 | 1.34E-03 |
| proteinaceous extracellular matrix (GO:0005578) | 229 | 17 | 4.48 | + | 3.8 | 3.69E-03 |
| extracellular matrix (GO:0031012) | 293 | 21 | 5.73 | + | 3.66 | 4.68E-04 |
| synapse part (GO:0044456) | 240 | 16 | 4.69 | + | 3.41 | 2.60E-02 |
| plasma membrane region (GO:0098590) | 377 | 23 | 7.37 | + | 3.12 | 2.02E-03 |
| synapse (GO:0045202) | 352 | 21 | 6.88 | + | 3.05 | 7.87E-03 |
| cell surface (GO:0009986) | 389 | 23 | 7.61 | + | 3.02 | 3.36E-03 |
| membrane region (GO:0098589) | 461 | 27 | 9.02 | + | 2.99 | 5.57E-04 |
| intrinsic component of plasma membrane (GO:0031226) | 718 | 38 | 14.04 | + | 2.71 | 3.37E-05 |
| extracellular space (GO:0005615) | 656 | 34 | 12.83 | + | 2.65 | 3.03E-04 |
| plasma membrane part (GO:0044459) | 1265 | 62 | 24.74 | + | 2.51 | 1.73E-08 |
| cell junction (GO:0030054) | 656 | 32 | 12.83 | + | 2.49 | 2.45E-03 |
| integral component of plasma membrane (GO:0005887) | 687 | 32 | 13.44 | + | 2.38 | 6.25E-03 |
| neuron part (GO:0097458) | 605 | 28 | 11.83 | + | 2.37 | 2.71E-02 |
| cell periphery (GO:0071944) | 2400 | 96 | 46.94 | + | 2.05 | 1.63E-09 |
| cell projection (GO:0042995) | 926 | 37 | 18.11 | + | 2.04 | 3.32E-02 |
| plasma membrane (GO:0005886) | 2327 | 92 | 45.51 | + | 2.02 | 1.13E-08 |
| extracellular region part (GO:0044421) | 2338 | 87 | 45.73 | + | 1.9 | 1.13E-06 |
| extracellular region (GO:0005576) | 2562 | 93 | 50.11 | + | 1.86 | 8.44E-07 |
| extracellular vesicle (GO:1903561) | 1774 | 63 | 34.7 | + | 1.82 | 2.10E-03 |
| extracellular exosome (GO:0070062) | 1774 | 63 | 34.7 | + | 1.82 | 2.10E-03 |
| extracellular organelle (GO:0043230) | 1776 | 63 | 34.74 | + | 1.81 | 2.18E-03 |
| extracellular membrane-bounded organelle (GO:0065010) | 1776 | 63 | 34.74 | + | 1.81 | 2.18E-03 |
| membrane-bounded vesicle (GO:0031988) | 2099 | 72 | 41.05 | + | 1.75 | 1.15E-03 |
| vesicle (GO:0031982) | 2171 | 73 | 42.46 | + | 1.72 | 1.97E-03 |
| intrinsic component of membrane (GO:0031224) | 2454 | 82 | 48 | + | 1.71 | 4.64E-04 |
| membrane part (GO:0044425) | 3212 | 105 | 62.82 | + | 1.67 | 1.45E-05 |
| integral component of membrane (GO:0016021) | 2397 | 77 | 46.88 | + | 1.64 | 5.24E-03 |
| membrane (GO:0016020) | 4998 | 139 | 97.76 | + | 1.42 | 5.71E-04 |
| cellular_component (GO:0005575) | 11241 | 267 | 219.86 | + | 1.21 | 1.03E-07 |
| cell (GO:0005623) | 9779 | 227 | 191.27 | + | 1.19 | 1.00E-02 |
| cell part (GO:0044464) | 9731 | 225 | 190.33 | + | 1.18 | 1.87E-02 |
| intracellular membrane-bounded organelle (GO:0043231) | 6248 | 87 | 122.21 | - | 0.71 | 1.73E-02 |
| intracellular organelle part (GO:0044446) | 4341 | 54 | 84.91 | - | 0.64 | 2.47E-02 |
| nucleus (GO:0005634) | 3921 | 37 | 76.69 | - | 0.48 | 1.30E-05 |
| Unclassified (UNCLASSIFIED) | 4455 | 41 | 87.14 | - | 0.47 | 0.00E+00 |
| nuclear part (GO:0044428) | 2376 | 16 | 46.47 | - | 0.34 | 3.73E-05 |
| intracellular organelle lumen (GO:0070013) | 2259 | 12 | 44.18 | - | 0.27 | 1.43E-06 |
| organelle lumen (GO:0043233) | 2263 | 12 | 44.26 | - | 0.27 | 1.34E-06 |
| membrane-enclosed lumen (GO:0031974) | 2292 | 12 | 44.83 | - | 0.27 | 8.19E-07 |
| nuclear lumen (GO:0031981) | 2102 | 10 | 41.11 | - | 0.24 | 1.13E-06 |
| nucleoplasm (GO:0005654) | 1651 | 7 | 32.29 | - | 0.22 | 2.65E-05 |
|  |  |  |  |  |  |  |
| **GO molecular function complete** |  |  |  |  |  |  |
| titin binding (GO:0031432) | 7 | 4 | 0.14 | + | > 5 | 2.33E-02 |
| quaternary ammonium group binding (GO:0050997) | 27 | 6 | 0.53 | + | > 5 | 3.33E-02 |
| cytoskeletal protein binding (GO:0008092) | 569 | 31 | 11.13 | + | 2.79 | 6.95E-04 |
| receptor binding (GO:0005102) | 795 | 43 | 15.55 | + | 2.77 | 3.84E-06 |
| calcium ion binding (GO:0005509) | 467 | 25 | 9.13 | + | 2.74 | 1.31E-02 |
| protein binding (GO:0005515) | 3733 | 123 | 73.01 | + | 1.68 | 3.42E-07 |
| binding (GO:0005488) | 7818 | 197 | 152.91 | + | 1.29 | 5.00E-04 |
| molecular_function (GO:0003674) | 10621 | 247 | 207.74 | + | 1.19 | 7.26E-04 |
| Unclassified (UNCLASSIFIED) | 5075 | 60 | 99.26 | - | 0.6 | 0.00E+00 |
| nucleic acid binding (GO:0003676) | 2456 | 18 | 48.04 | - | 0.37 | 2.05E-04 |
